# Quantitative tools and measurements for assessing the implementation of regulatory policies in reducing alcohol consumption and alcohol‐related harms: A scoping review

**DOI:** 10.1111/dar.13543

**Published:** 2022-09-12

**Authors:** Jintana Jankhotkaew, Sally Casswell, Taisia Huckle, Surasak Chaiyasong, Payao Phonsuk

**Affiliations:** ^1^ SHORE & Whariki Research Centre Massey University Auckland New Zealand; ^2^ International Health Policy Program Ministry of Public Health Nonthaburi Thailand; ^3^ Alcohol and Health Promotion Policy Research Unit and Social Pharmacy Research Unit, Faculty of Pharmacy Mahasarakham University Maha Sarakham Thailand; ^4^ Department of Health Education and Behavioral Sciences, Faculty of Public Health Mahidol University Bangkok Thailand

**Keywords:** alcohol policy, policy implementation, quantitative measurement, scoping review, tool

## Abstract

**Issues:**

Implementation of alcohol control policy is a global priority as alcohol contributes to negative individual health and societal impacts. However, there are no available reviews that comprehensively provide tools and measurements for assessing the implementation of alcohol control policy. This study reviews tools and measurements for assessing alcohol policy implementation. Policies considered include alcohol pricing and taxation, alcohol marketing control, physical availability control and drink‐driving policy.

**Approach:**

We conducted a scoping review from Scopus, Web of Science and the World Health Organization's website. We included studies on policy implementation for the four most effective prevention policies published worldwide between 2000 and 2021.

**Key Findings:**

The search yielded 11,654 articles and these were narrowed down to 39 included studies. Of these 39 studies, almost half assessed the implementation of a drink‐driving policy (*n* = 19), followed multipolicy (*n* = 12) and physical availability control (*n* = 8). There was no single study assessing policy implementation of pricing and taxation or alcohol marketing control. The majority of the studies were conducted in high‐income countries (*n* = 31). Globally, there is no standardised tool or guidelines for measuring the policy implementation of these four policies. The tools for measuring policy implementation mostly focused on a single policy, and few covered multiple policies.

**Implications:**

We recommend developing standardised tools and measurements to monitor policy implementation across multiple policies at country levels.

**Conclusion:**

This review highlighted a lack of comprehensive and standardised tools to assess policy implementation and the limited number of studies on alcohol policy implementation in low‐ and middle‐income countries.

**Key Points**

- This review is the first to systematically review quantitative tools for assessing alcohol policy implementation across effective alcohol policies.
- This review highlights the lack of standardised tools to monitor policy implementation.
- The findings contribute to policy implementation by collating available tools worldwide and addressing the comprehensiveness of the tools from a policy implementation perspective.

## 1 INTRODUCTION

Alcohol consumption has negative impacts on individual health and on society. Alcohol consumption contributes to more than 230 diseases and injuries such as liver cancer, tuberculosis, foetal alcohol spectrum disorders, alcohol dependence and suicide. Globally, there are six deaths every minute from alcohol‐related diseases and injuries [1]. Not only this, but alcohol also has serious negative impacts on society as a whole, such as contributing to domestic violence and crime. In addition, alcohol consumption has resulted in productivity and economic losses, ranging from 0.45% to 5.44% of gross domestic product [2].

Policy implementation refers to transferring policies into practice. It is mainly focused on the processes of implementation (carrying out, accomplishing, fulfilling, producing and completing policy goals) [3]. Various domains that are considered important, including the content of policies or characteristics of interventions, factors within implementing agencies (e.g., resources, skills, the acceptability of the policy to the implementing agencies), contextual factors (political, social, cultural factors) [4, 5]. Implementation processes include training, coordinating, raising public awareness and law enforcement [6]. Also, immediate results and short‐term outcomes (e.g., knowledge about policies or law among implementers and general population, perceived of law enforcement among general population), are useful as they help to identify steps and progress in the policy implementation process.

Despite increasing evidence of the effectiveness of alcohol control policies, particularly the so‐called best‐buy policies [7], there has been less attention on translating those policies into practice in various settings, particularly in low‐ and middle‐income countries. In addition, alcohol policy implementation is challenging, as efforts and commitments are beyond the health sector and include the context of powerful commercial interests [8]. The complexity of policy implementation requires sound measurements and standardised tools for measuring alcohol policy implementation to track progress and prioritise policy actions.

Measuring policy implementation is an essential step for identifying the progress of policy implementation at national and global levels. Quantitative measures and tools can help to measure the progress of policy implementation and identify potential facilitators or barriers that affect policy implementation outcomes and can be designed to be comparative across countries. To allow countries to monitor their progress requires tools to enable comprehensive measurements of policy content, implementation processes and policy implementation context [9] and to monitor policy implementation in different policy areas. This can help countries identify implementation gaps and effectively prioritise their actions and efforts to invest limited resources efficiently. As a result, it can help countries leverage and accelerate alcohol policy implementation for achieving the Sustainable Development Goal 3.5.2, reducing alcohol consumption per capita.

Globally, few tools have been developed to measure the progress of policy implementation at the national level [10, 11, 12]. Most of the available studies focused on the comprehensiveness and restrictiveness of policies [13, 14, 15, 16]. Few of them included measurements of policy implementation. One example which included implementation is the Alcohol Prevention Magnitude Measure, which was developed to measure policy implementation in Sweden [12]; however, this tool has not been tested elsewhere. As most available tools focus on the comprehensiveness and restrictiveness of policies, they might not be able to capture the complexity of the factors that influence the implementation outcomes in different settings regarding social, economic, political and cultural aspects; the outcome of implementation depends on more than just an effective intervention. Other aspects include cultural aspects and social norms, political commitment, and the capacity and motivation of the implementing agency itself. Hence, a systematic review of tools for assessing the implementation of effective policies can help generate tools that capture the overall complexity of the implementation process.

At present, no systematic review or scoping review has been conducted to identify tools and measurements for assessing the implementation of alcohol control policy. Hence, this review is intended to fill this gap by focusing on the three ‘best buy policies’: alcohol pricing and taxation, control of alcohol marketing, control of physical availability and one effective intervention, on drink‐driving [7]. This review aims to identify tools and measurements for assessing alcohol policy implementation.

## 2 METHODS

We conducted a scoping review following the approach of the Joanna Briggs Institute (JBI) [17] and registered the protocol through the Open Science Framework. The JBI's guideline has been widely used and cited worldwide [18]. A scoping review was deemed an appropriate choice for this review to explore the breadth and depth of literature, map existing knowledge and identify gaps of knowledge [19]. A scoping review does not require quality assessment or a critical appraisal of the included studies and its' purpose is not to critically review tools, their content or application.

To ensure the accuracy of the scoping review, we followed the six approaches to conduct a scoping review provided by JBI [17].

### 2.1 Stage 1: Identifying the research questions and scope of the study

The scoping review aimed to explore the evidence on tools and measurements available to assess the implementation of effective alcohol control policies. We developed a research question regarding the gap in evidence on the implementation of alcohol control policy. What tools and measurements have been used to assess the implementation of regulatory policies to reduce alcohol consumption and alcohol‐related harm? We decided to focus on four regulatory measures with the strongest evidence base for effectiveness: alcohol taxation and pricing, control of marketing, control of physical availability and drink‐driving measures [7]. The operational definitions of the terms used in this review are as follows. Alcohol policy included any regulatory measures implemented at the national level or sub‐national levels. The alcohol policies of interest included pricing and taxation policies, alcohol marketing control, physical availability control and drink‐driving policy. Physical availability included regulating retail outlets, densities of retail outlets, restricting hours and days of trade, ban on public drinking, minimum purchasing age, licensing, control of social supply and online sales [20]. Alcohol marketing covers any regulatory measures that control any forms of alcohol marketing (i.e., alcohol advertisement, promotion, pricing promotion, alcohol sponsorship, products and placement) [20].

Policy implementation means carrying out, accomplishing, fulfilling, producing and completing policy goals [3]. We focused on the following key areas: first, factors inside implementing agencies included available resources (e.g., financial, materials and human resources) [4, 5]. Second, policy implementation process including activities to be carried out to implement alcohol control policies (e.g., coordinating between implementing agencies, education, raising public awareness, training and law enforcement) [6]. Third, short‐term outcomes of policy implementation made by governments (e.g., knowledge about law content among implementers and general population, perception towards law enforcement among general population). This review does not focus on measurement of the intermediate‐term outcomes (e.g., alcohol consumption patterns) and impacts (i.e., alcohol‐related harm) which has been covered by a guideline from the World Health Organization (WHO) 2000 [21]. Fourth, the context of policy implementation was also a focus (e.g., social, cultural, economic and political contexts). Last, characteristics of interventions or policy content, which focused on investigating the restrictiveness and stringency of policies and implementation were included (i.e., we excluded studies that focused only on policy content).

A quantitative tool refers to any tools used to collect data for measuring policy implementation and outcomes of policy implementation. Measurement is the process of an operationalised abstract construct (i.e., policy implementation) into concrete variables [22, 23] in order to determine policy implementation processes and outcomes.

### 2.2 Stage 2: Developing and conducting a search strategy

We developed a search strategy in Scopus and Web of Science. We selected Scopus as it is the largest search engine of scientific literature [24], including 100% of MEDLINE health science topics. We selected Web of Science, which covered some of the articles not covered in the Scopus. We also included grey literature from WHO website. A search strategy was developed for Scopus and revised appropriately for Web of Science and WHO's website. The key search strategy can be found in Table S1, Supporting Information. The last date that we conducted the search strategy was 18 May 2021.

#### 2.2.1 Inclusion criteria

Inclusion criteria consisted of three aspects regarding context, concept and population. All included studies needed to meet the three criteria. First, the context, we included any literature from any setting. Second, the concepts, we included relevant studies that addressed quantitative tools or measures for assessing the implementation of one of the four policies: alcohol taxation and pricing, control of marketing, control of physical availability and drink‐driving policy. The studies included in this review focused on policy implementation regarding the scope mentioned earlier. Third, the populations, we included any population group.

This review included publications published between 2000 and 2021 to ensure up‐to‐date evidence. We included published and grey literature. The grey literature included government reports and technical reports. Quantitative and mixed‐methods were included to ensure that we included studies that used tools and quantitative measurements for assessing the implementation of alcohol control policies. We included primary and secondary research that quantified policy implementation.

#### 2.2.2 Exclusion criteria

We excluded various types of studies. First, studies of policy implementation that did not investigate the four policies listed above. Second, we excluded any studies that did not provide tools or measurements. Third, we excluded any literature that was not written in English. Last, we excluded any studies that only applied qualitative methods.

### 2.3 Stage 3: Evidence screening and selection

Two reviewers (JJ and PP) independently screened titles and abstracts following the review protocol. The full texts of studies selected for inclusion were then screened. In the case of a small number of disagreements, they were discussed and a consensus was achieved.

### 2.4 Stage 4: Data extraction

Based on the previous template suggested by the JBI [25], a data extraction form was developed and adapted during the protocol setting stage and piloted and adjusted during the review stage. The data extraction form was designed according to the research questions and objectives. It included authors, study country, objectives of the study, policy levels, settings, policy areas, design and methods, tools and key components of measurements assessing policy implementation. Prior to the use of the data extraction form, two reviewers independently tested the data extraction form and discussed improvements to the comprehensiveness and clarity of the form. The agreements were based on consensus between the two reviewers. The full data extraction process was done by one independent reviewer (JJ) and one reviewer (PP) verified the data for accuracy for every included study. If there were inconsistencies, the decisions were based on the consensus between the two reviewers.

### 2.5 Stage 5: Data analysis

The purpose of this scoping review is to map and aggregate findings on the tools that existing studies have used for assessing the implementation of government policies. The data extracted from the studies were analysed descriptively using Stata 16. We categorised components and measurements in alignment with adapted domains from a logic model and policy implementation domains, namely policy content, inputs (e.g., resources and workforce), processes of policy implementation (e.g., cooperation, enforcement and publicising enforcement activities) and short‐term outcomes (e.g., compliance with laws, perception towards law enforcement, knowledge about law content) and context of policy implementation [4, 9, 26]. This review investigated aspects of comprehensiveness in terms of tools in two dimensions: policy areas and policy domains (law content, inputs, process, short‐term implementation outcomes and context). If there is a tool mentioned in the study, we also investigated whether its reliability and validity have been assessed (see definitions in Table 1).

**TABLE 1:** Validity and reliability domains and definition

Domain	Definition
Validity	The instrument measured what it intends to measure.
Content validity	Experts in the discipline scrutinise the instrument very carefully and make value judgements regarding construct.
Criterion‐related validity	Comparing scores obtained from the instrument with external instrument (i.e., gold standard) by investigating correlation coefficient between results of the instruments.
Construct validity	Determining one construct is correlated with another relevant construct that is being measured, but not the gold standard.
Known‐group technique	Instruments can differentiate outcomes that vary across different population.
Reliability	Internal consistency of instruments.

*Source*: Thomas [23].

### 2.6 Stage 6: Presentation of the results

We followed the preferred reporting items for systematic review and meta‐analysis extension for scoping review (PRISMA‐ScR) checklist (see Table S2, Supporting Information).

## 3 RESULTS

### 3.1 Search results

**FIGURE 1:**
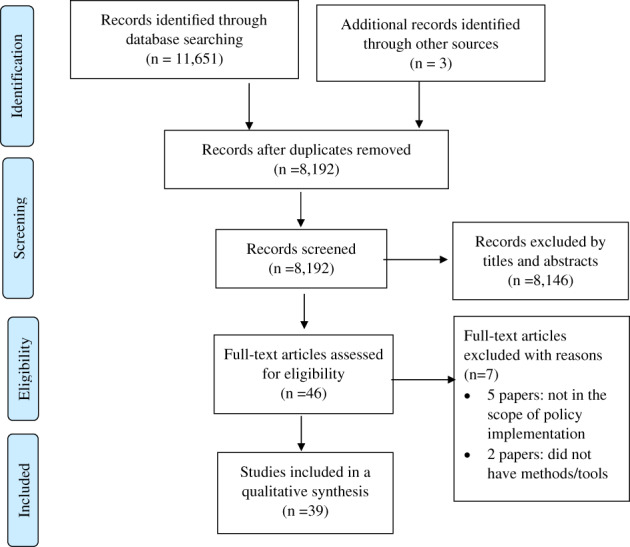
Article screening process

A total of 11,654 papers were identified from the databases and an additional search from WHO's website. After removing duplication, we screened 8,192 titles and abstracts. After excluding papers that were not in the scope of this review, we had 46 to assess for eligibility. We excluded seven articles for the following reasons: five papers were not in the scope of policy implementation, mainly focused on the restrictiveness of policies, and two papers did not provide sufficient details on tools or measurements (Figure 1).

### 3.2 Characteristics of the selected studies

Thirty‐one studies were conducted in high‐income countries, three were conducted in low‐ and middle‐income countries, and five were multi‐country. Regarding study design, the majority used cross‐sectional surveys (*n* = 18), multisource data (*n* = 14) and mixed methods (*n* = 7). For policy areas, almost half of the studies were primarily focused on drink‐driving policy (*n* = 19) and physical availability control (*n* = 8). Twelve studies investigated the implementation of alcohol policies across different policy areas. No study examined tools that captured the implementation of pricing and taxation policy and alcohol marketing control. Under physical availability control, most of them were minimum purchasing age (*n* = 6) (Table 2).

**TABLE 2:** Characteristics of the selected studies

Characteristics of the selected studies	Total number of studies, *n* = 39 (%)
Country	
High‐income countries	31 (79)
USA	23 (59)
New Zealand	2 (5)
Sweden	2 (5)
Australia (1), Chile (1), Spain (1) and UK (1)	4 (10)
Low‐ and middle‐income countries	3 (8)
China	2 (5)
Cambodia	1 (3)
Multiple countries	5 (13)
Study design	
Cross‐sectional study	18 (46)
Multi‐source data	14 (36)
Mixed method	7 (18)
Level of study	
National level	21 (54)
Sub‐national level	13 (33)
Multi‐countries	5 (13)
Policy areas	
Physical availability control policy (minimum purchasing age (6), licensing (1), social supply to underage (1))	8 (20)
Drink‐driving policy	19 (49)
Combining at least two policy areas	12 (31)

### 3.3 Tools for assessing the policy implementation

We found that there is no standardised tool available for assessing alcohol policy implementation among the four policies. Among the studies that applied tools, most of them used questionnaires (*n* = 32). However, only four studies assessed the validity of the questionnaires. One study assessed content validity and another study applied content validity and criterion‐related validity. The study that assessed criterion‐related validity was conducted in nine countries in the Western Pacific Region by investigating the association between alcohol policy score and alcohol consumption per capita [11]. Furthermore, two studies applied the known‐group technique. Those two studies were conducted in Sweden and generated the Alcohol Prevention Magnitude Measure (APMM), which measured the progress of policy implementation. The studies found that the APMM varied across communities with interventions and without interventions [12, 27]. In total, only five studies assessed the reliability of the tool.

In addition, the majority of studies applied a tool assessing a single policy, and we found that only two studies provided a tool for assessing implementation across the four‐policy areas. These two studies included the International Alcohol Control Study, which included a component on the assessment of alcohol policy implementation using stringency and impact via surveys of, for example, price [10, 28]. Another study was the Toolkit for Evaluating Alcohol Policy Stringency and Enforcement‐16 (TEASE‐16), which measured policy enforcement by reviewing policy enforcement from available data and government reports [11]. In addition, the tool APMM, generated and tested in Sweden, quantified the implementation of the three main policies: physical availability, brief intervention and drink‐driving measures. The APMM was tested for reliability and the components of APMM were mainly focused on resources, network and communications as well as activities that carry out to implement policies [12].

### 3.4 Components of measurements for assessing policy implementation among the four policies

Overall, regarding components of measurements for assessing the policy implementation, the majority of the studies assessed the context of policy implementation (*n* = 29) and processes of policy implementation (*n* = 29), followed by short‐term outcomes of policy implementation (*n* = 16), inputs (*n* = 14) and law content (*n* = 5) (Table 3).

**TABLE 3:** Components of measurements for quantifying policy implementation and short‐term outcomes of implementation outcomes of four policies

Sets of key components	A brief description and example of relevant measurement	Policies (*n*)	Total, *n* (%)	Included studies [references]
Policy content			5 (13)	
Stringency and effectiveness of alcohol legislation/regulation	Stringency and effectiveness of alcohol legislation/regulation using policy scores, constructed from a level of stringency and effectiveness rating.	Availability (1) and multi‐policies (4)	5 (13)	[10, 11, 29, 30, 31]
Input			14 (36)	
Available resources	Presence/absence of implementers or implementing agencies Numbers of full‐time officers per capita. Numbers of officers responsible for law enforcement per capita. Having specific unit assigned for law enforcement related to alcohol (yes/no).	Availability (2), drink‐driving (5) and multi‐policies (4)	11 (28)	[12, 27, 32, 33, 34, 40, 41, 42, 43, 44, 45]
	Resources available for implementation Fund of enforcement activities per capita. Percentage of total annual resources in agencies devoted to enforce drink‐driving laws.	Drink‐driving (2) and multi‐policies (4)	6 (15)	[12, 27, 29, 30, 35, 42]
Implementation process			29 (74)	
Executing	Cooperation with key stakeholders Organising cooperation with relevant authorities (yes/no).	Multi‐policies (2)	2 (5)	[12, 27]
	Efforts on law enforcement, including presence/absence and intensity of law enforcement Conducting enforcement activities (yes/no). Frequency of enforcement. Level of enforcement (Likert scales). Number of driving under the influence of alcohol arrests (proxy of enforcement). Number of sobriety checkpoints/annual number of traffic stops per capita/frequency of sobriety checkpoints (weekly, monthly, less than monthly, never). Proportion of inspected grocery shops selling alcohol.	Availability (5), and drink‐driving (14) and multi‐policies (8)	27 (69)	[10, 11, 12, 27, 31, 32, 33, 34, 35, 40, 42, 43, 44, 45, 46, 47, 48, 49, 50, 51, 52, 53, 54, 55, 56, 57, 58]
	Publicity of law enforcement and raising awareness of alcohol Publicity of findings of law enforcement (yes/no). Active work with media advocacy to increase awareness about alcohol (yes/no).	Drink‐driving (3) and multi‐policies (2)	5 (13)	[12, 27, 48, 49, 57]
Implementation outcomes: short‐term outcomes			16 (41)	
Knowledge about laws among implementers and the general population	Knowledge about laws among implementers and general population Knowledge on laws (true/false and Likert scale). Police's perception towards their skill and competence (Likert scale).	Availability (1) and drink‐driving (3)	4 (10)	[50, 51, 59, 60]
Deterrent effect	Deterrent effect and perceptions towards law enforcement Perceived law enforcement (Likert scales). Perceived likelihood of being caught (Likert scales). Perceived likelihood of being arrested if the police stopped (Likert scales). Perceptions towards punishment (Likert scales).	Availability (3), drink‐driving (4), multi‐policies (4)	11 (28)	[28, 29, 30, 54, 59, 61, 62, 63, 64, 65, 66]
Compliance to law	Compliance with alcohol law Percentage of respondents who violate alcohol control law. Level of compliance (Likert scale).	Availability (1) and multi‐policies (2)	3 (8)	[10, 50, 56]
Contextual factors			29 (74)	
Characteristics of implementers who involved in the policy implementation	Characteristics of implementers Gender, age, position or rank in service, number of years were in service.	Drink‐driving (1)	1 (3)	[50]
Social, cultural and economic context	Characteristics of general population (age, gender, race, household income, marital status, ethnicity and education level).	Availability (3), drink‐driving (8) and multi‐policies (4)	15 (38)	[28, 29, 30, 31, 34, 51, 52, 54, 57, 59, 61, 62, 63, 65, 66]
	Social norms on alcohol drinking, alcohol‐related harm and perception towards alcohol availability Perception towards alcohol use (Likert scales). Perceived alcohol harm (Likert scales). Perceived acceptability of alcohol use (Likert scales). Perception towards drink‐driving behaviours (Likert scales). Perceived ease of alcohol availability (Likert scales).	Availability (3), drink‐driving (3) and multi‐policies (5)	11 (28)	[28, 29, 30, 42, 44, 45, 61, 62, 63, 65, 66]
	Measures on contexts of community or state context include two aspects: alcohol‐related context and demographic/social/economic context. Alcohol‐related context. Drinking norm (Wet, moderate and dry). Outlet density or outlets per roadway mile in each city. Demographic/social/economic characteristics Poverty rate. Percent of ethnicity. Population. Unemployment rates. Religiosity. Deprivation index.	Availability (5), drink‐driving (9), multi‐policies (5)	19 (49)	[40, 46, 47] [27, 29, 30, 31, 32, 33, 35, 41, 42, 43, 44, 45, 50, 52, 54, 58]

Regarding the context of policy implementation, most of the studies assessed community characteristics (*n* = 19) (e.g., outlet density, poverty rates, percent of ethnicity, unemployment rates) and individual characteristics (*n* = 15) (e.g., age, gender, race, household income). Community characteristics and individual characteristics were mainly included in the studies on drink‐driving policy, physical availability control and multi‐policy. Beside this, 11 studies investigated social norms such as perception towards alcohol use, alcohol availability and alcohol‐related harm (Table 3).

Regarding the process of policy implementation, the majority of the studies investigated the presence/absence and intensity of law enforcement (*n* = 27). Most of these studies investigated drink‐driving policy (*n* = 14) and multi‐policy (*n* = 8). Five studies investigated the presence/absence of publicity of enforcement and media advocacy for raising awareness (Table 3).

Regarding the short‐term outcome components, the majority of the studies investigated short‐term outcomes, including the deterrent effect of law enforcement (*n* = 11). The majority of studies investigating the deterrent effect of law primarily focused on drink‐driving (*n* = 4) and multi‐policy (*n* = 4) (Table 3).

Regarding inputs of policy implementation, most of the studies investigated the existence of implementers and implementing agencies (*n* = 11). In addition, six studies investigated resources allocated for policy implementation. The majority of these studies were found among multiple policies (*n* = 4) and drink‐driving policy (*n* = 2) (Table 3).

Regarding law content, most of the studies investigated the restrictiveness and effectiveness of alcohol regulation, and among those were multi‐policy studies (*n* = 4) (Table 3).

Considering comprehensive studies regarding measurements, we found that none of the included studies investigated the implementation of a policy in all aspects, including content, input, process, short‐term outcomes and context. Eight studies covered four components of implementation [27, 29, 30, 31, 32, 33, 34, 35]. Among these studies, three of them investigated policy implementation across different policies [27, 29, 30]. See more details of the findings of the included studies in Table S3, Supporting Information.

## 4 DISCUSSION

This review is the first study that comprehensively reviewed tools and measurements for assessing alcohol policy implementation. This review contributes to the research community in mapping the published tools or measurements available worldwide. This study found no standardised tools or guidelines for measuring policy implementation among the four policy areas. In addition, there were few studies that assessed the validity and reliability of the tools. However, the measurement components for assessing implementation in this review reflect a wide array of policy implementation aspects, including law content, implementation inputs, implementation processes, short‐term implementation outcomes and implementation context. Overall, the literature is dominated by the studies on the process of law enforcement effort, including the presence and absence and intensity of law enforcement.

Most studies assessed the implementation of a single policy, even though more than one type of alcohol policy is usually implemented in a country. Also, studies tend to focus on just some aspects of implementation. To assess how well policy is implemented, we recommend that a more comprehensive approach be taken where multiple effective policies are measured along with a range of key implementation measures. Using a more comprehensive approach and tools will allow for both wider‐assessment of how well policies are implemented at a country‐level and for how well a country is doing for each type of policy, both of which can help countries prioritise their actions effectively.

When considering comprehensive studies found in this review, few studies investigated a range of policy implementation domains. As policy implementation is an interactive process [36], comprehensive studies (i.e., studies that investigate all aspects of policy domains and across multiple policies) are required to help countries prioritise their actions effectively.

We did not find a study using tools or measurements to assess policy implementation of alcohol marketing and pricing policy. The findings may reflect the policy situation globally. First, there are few countries that have implemented regulatory measures to control alcohol marketing [37]. Second, most of the studies on pricing and taxation policy focused on effectiveness, particularly impact on alcohol consumption (i.e., price elasticity) [38], which we did not include in this review. The International Alcohol Control Policy Index which included a measure of the impact of policies, intended to reflect implementation, found measures of both price and marketing were associated with per capita consumption across several countries [39].

### 4.1 Implications

It is important to quantify policy implementation to evaluate its progress. Quantifying policy implementation (i.e., policy content, policy implementation process, outputs and short‐term implementation outcomes, context) helps to identify potential factors that influence outcomes of policy implementation as well as assess the progress of policy implementation. Later, it contributes to evidence‐based decision making to accelerate policy implementation. Our review collates tools and measurements available worldwide and reports the validity and reliability of the tools, where available. This comprehensive review of the tools can provide tools and measurements for countries to apply in their context to measure their policy implementation progress.

### 4.2 Limitations

A few limitations need to be addressed in this review. First, we did not include non‐English studies. Second, we assessed the validity and reliability of the tools based on the information provided by the included studies. However, authors may assess the validity and reliability of the study but not mention it in the published studies. To comprehensively assess the quality of tools, one could contact authors to ensure the accuracy of the information; however, this is not required in a scoping review. Third, this review only focused on four policy areas and regulatory policy. Therefore, it may not cover alcohol policy implementation overall but concentrate on those known to be most effective. Last, our review focused on specific alcohol policies and policy implementation in general, but did not apply the concept or definition of policy implementation from the political science nor implementation science more specifically in the search strategy.

## 5 CONCLUSION

This review highlighted that there is no standardised tool for measuring alcohol policy implementation. Among available tools, there are few that assessed the validity and reliability of the tools. When considering measurement aspects, there is a lack of studies investigating the whole range of alcohol policy implementation domains, from policy content, policy inputs, policy implementation processes, short‐term outcomes of policy implementation and policy context, and few studies assessed multiple policies simultaneously. Moreover, research on tools and measurements for assessing policy implementation is lacking in low‐ and middle‐income country contexts; therefore, more research is needed to enhance policy implementation in these contexts.

## AUTHOR CONTRIBUTIONS

Each author certifies that their contribution to this work meets the standards of the International Committee of Medical Journal Editors.

## FUNDING INFORMATION

This work was supported by Thai Health Promotion Foundation under the Strengthening Networks and Technical Capacity for Alcohol Policy Development under the WHO‐ThaiHealth Memorandum of Understanding on Health Promotion 2018‐2020 (grant numbers 61‐00‐1928) and the Capacity Building on Health Policy and Systems Research programme (HPSR Fellowship) under cooperation between National Health Security Office, Bank for Agriculture and Agricultural Co‐operatives, and International Health Policy Programme Foundation. The authors also gratefully acknowledge the funding support through project of Public Policies, Law, and Non‐communicable Diseases in Thailand: A Policy Implementation and System Research by International Health Policy Programme from the Thailand Science Research and Innovation. The funders have no role in the study design; collection, analysis and interpretation of data; writing of the study report, or decision to submit for publication.

## CONFLICT OF INTEREST

None to declare.

## Supporting information


**Table S1** Search terms across different databases

**Table S2** Preferred reporting items for systematic reviews and meta‐analyses extension for scoping reviews (PRISMA‐ScR) checklist

**Table S3** Summary of included tools and measurements for assessing policy implementation across four policies

## References

[1] World Health Organization . Global status report on alcohol and health 2018. Geneva: World Health Organization; 2018.

[2] Thavorncharoensap M , Teerawattananon Y , Yothasamut J , Lertpitakpong C , Chaikledkaew U . The economic impact of alcohol consumption: a systematic review. Subst Abuse Treat Prev Policy. 2009;4:20. 19939238 10.1186/1747-597X-4-20 PMC2791094

[3] Pressman JK , Wildavsky A . Implementation: how great expectations in Washington are dashed in Oakland. 3rd ed. Berkeley: University of California Press; 1984.

[4] Breimaier HE , Heckemann B , Halfens RJG , Lohrmann C . The consolidated framework for implementation research (CFIR): a useful theoretical framework for guiding and evaluating a guideline implementation process in a hospital‐based nursing practice. BMC Nurs. 2015;14:43. 26269693 10.1186/s12912-015-0088-4 PMC4533946

[5] Damschroder LJ , Aron DC , Keith RE , Kirsh SR , Alexander JA , Lowery JC . Fostering implementation of health services research findings into practice: a consolidated framework for advancing implementation science. Implement Sci. 2009;4:50. 19664226 10.1186/1748-5908-4-50 PMC2736161

[6] Jones‐Webb R , Nelson T , McKee P , Toomey T . An implementation model to increase the effectiveness of alcohol control policies. Am J Health Promot. 2014;28:328–35. 23971519 10.4278/ajhp.121001-QUAL-478

[7] World Health Organization . Best buys' and other recommended interventions for the prevention and control of noncommunicable diseases: updated (2017) appendix 3 of the global action plan for the prevention and control of noncommunicable diseases 2013–2020. Geneva: World Health Organization; 2017.

[8] World Health Organization . Follow‐up to the high‐level meetings of the United Nations general assembly on health‐relaed issues: political declaration of the third high‐level meeting of the general assembly on the prevention and control of non‐communicable diseases: findings of the consultative process on implementation of the global strategy to reduce the harmful use of alcohol and the way forward. Geneva: World Health Organization; 2019.

[9] Hogwood BW , Gunn AL . Policy analysis for the real world. New York: Oxford University Press; 1984.

[10] Casswell S , Morojele N , Williams PP , Chaiyasong S , Gordon R , Gray‐Phillip G , et al. The alcohol environment protocol: a new tool for alcohol policy. Drug Alcohol Rev. 2018;37(Suppl 2):S18–26. 29314356 10.1111/dar.12654 PMC6208285

[11] Carragher N , Byrnes J , Doran CM , Shakeshaft A . Developing an alcohol policy assessment toolkit: application in the western Pacific. Bull World Health Organ. 2014;92:726–33. 25378726 10.2471/BLT.13.130708 PMC4208478

[12] Nilsson T , Leifman H , Andréasson S . Monitoring local alcohol prevention in Sweden: application of alcohol prevention magnitude measure (APMM). NAD Nord Stud Alcohol Drugs. 2015;32:479–94.

[13] Madureira‐Lima J , Galea S . Alcohol control policies and alcohol consumption: an international comparison of 167 countries. J Epidemiol Community Health. 2018;72:54–60. 29061844 10.1136/jech-2017-209350

[14] Erickson DJ , Lenk KM , Toomey TL , Nelson TF , Jones‐Webb R , Mosher JF . Measuring the strength of state‐level alcohol control policies. World Med Health Policy. 2014;6:171–86. 25574422 10.1002/wmh3.97 PMC4283838

[15] Naimi TS , Blanchette J , Nelson TF , Nguyen T , Oussayef N , Heeren TC , et al. A new scale of the U.S. alcohol policy environment and its relationship to binge drinking. Am J Prev Med. 2014;46:10–6. 24355666 10.1016/j.amepre.2013.07.015 PMC3878154

[16] Brand DA , Saisana M , Rynn LA , Pennoni F , Lowenfels AB . Comparative analysis of alcohol control policies in 30 countries. PLoS Med. 2007;4:e151. 17455992 10.1371/journal.pmed.0040151 PMC1876414

[17] Peters MDJ , Marnie C , Tricco AC , Pollock D , Munn Z , Alexander L , et al. Updated methodological guidance for the conduct of scoping reviews. JBI Evid Synth. 2020;18:2119–26. 33038124 10.11124/JBIES-20-00167

[18] Khalil H , Bennett M , Godfrey C , McInerney P , Munn Z , Peters M . Evaluation of the JBI scoping reviews methodology by current users. Int J Evid Based Healthc. 2020;18:95–100. 31567603 10.1097/XEB.0000000000000202

[19] Tricco AC , Lillie E , Zarin W , O'Brien K , Colquhoun H , Kastner M , et al. A scoping review on the conduct and reporting of scoping reviews. BMC Med Res Methodol. 2016;16:15. 26857112 10.1186/s12874-016-0116-4 PMC4746911

[20] Babor TF , Caetano R , Casswell S , Edwards G , Giesbrecht N , Graham K , et al. Alcohol: no ordinary commodity: research and public policy. 2010. 1–384.

[21] World Health Organization . International guide for monitoring alcohol consumption and related harm. Geneva: World Health Organization; 2000.

[22] Hagan TL . Measurements in quantitative research: how to select and report on research instruments. Oncol Nurs Forum. 2014;41:431–3. 24969252 10.1188/14.ONF.431-433

[23] Thomas RK . Quantitative nursing research. Thousand Oaks, Calif: SAGE Publications, Inc; 1998.

[24] Schotten M , Meester WJ , Steiginga S , Ross CA . A brief history of Scopus: the world's largest abstract and citation database of scientific literature. Research analytics: boosting university productivity and competitiveness through Scientometrics. New York: CRC Press; 2017.

[25] Aromataris E , Munn Z . JBI manual for evidence synthesis. 2020. Available from: https://synthesismanual.jbi.global.

[26] Funnell SC , Rogers PJ . Purposeful program theory: effective use of theories of change and logic models. 1st ed. Indianapolis: Jossey‐Bass; 2011.

[27] Nilsson T , Norström T , Andréasson S , Guldbrandsson K , Allebeck P , Leifman H . Effects of local alcohol prevention initiatives in Swedish municipalities, 2006‐2014. Subst Use Misuse. 2020;55:1008–20. 32024412 10.1080/10826084.2020.1720246

[28] Huckle T , Casswell S , Mackintosh AM , Chaiyasong S , Viet Cuong P , Morojele N , et al. The international alcohol control study: methodology and implementation. Drug Alcohol Rev. 2018;37:S10–7. 29292547 10.1111/dar.12650 PMC6120466

[29] Paschall MJ , Lipperman‐Kreda S , Grube JW . Effects of the local alcohol environment on adolescents' drinking behaviors and beliefs. Addiction. 2014;109:407–16. 24320952 10.1111/add.12397 PMC3945163

[30] Paschall MJ , Grube JW , Thomas S , Cannon C , Treffers R . Relationships between local enforcement, alcohol availability, drinking norms, and adolescent alcohol use in 50 California cities. J Stud Alcohol Drugs. 2012;73:657–65. 22630804 10.15288/jsad.2012.73.657 PMC3364331

[31] Erickson DJ , Lenk KM , Toomey TL , Nelson TF , Jones‐Webb R . The alcohol policy environment, enforcement and consumption in the United States. Drug Alcohol Rev. 2016;35:6–12. 26424225 10.1111/dar.12339 PMC4814360

[32] Nazif‐Munoz JI , Quesnel‐Vallée A , Van Den Berg A . Did Chile's traffic law reform push police enforcement? Understanding Chile's traffic fatalities and injuries reduction. Inj Prev. 2015;21:159–65. 25432938 10.1136/injuryprev-2014-041358

[33] Fell JC , Waehrer G , Voas RB , Auld‐Owens A , Carr K , Pell K . Effects of enforcement intensity on alcohol impaired driving crashes. Accid Anal Prev. 2014;73:181–6. 25240134 10.1016/j.aap.2014.09.002 PMC4254192

[34] Fell JC , Waehrer G , Voas RB , Auld‐Owens A , Carr K , Pell K . Relationship of impaired‐driving enforcement intensity to drinking and driving on the roads. Alcohol Clin Exp Res. 2015;39:84–92. 25515820 10.1111/acer.12598 PMC4308536

[35] Yao J , Johnson MB , Tippetts S . Enforcement uniquely predicts reductions in alcohol‐impaired crash fatalities. Addiction. 2016;111:448–53. 26451697 10.1111/add.13198

[36] Walt G . Health policy: an introduction to process and power. London: Zed Book Ltd.; 1994.

[37] Jernigan D . Global developments in alcohol policies: progress in implementation of the WHO global strategy to reduce the harmful use of alcohol since 2010. Geneva: World Health Organization; 2017.

[38] Nelson JP . Meta‐analysis of alcohol price and income elasticities ‐ with corrections for publication bias. Heal Econ Rev. 2013;3:17. 10.1186/2191-1991-3-17 PMC3722038 23883547

[39] Casswell S , Huckle T , Parker K , Romeo J , Graydon‐Guy T , Leung J , et al. Benchmarking alcohol policy based on stringency and impact: the international alcohol control (IAC) policy index. PLOS Glob Public Health. 2022;2:e0000109. 36962135 10.1371/journal.pgph.0000109 PMC10021514

[40] Calvert C , Toomey T , Lenk K , Joshi S , Nelson T , Erickson D . Variation in alcohol policy enforcement across urban and nonurban communities. J Rural Health. 2020;36:240–6. 31515854 10.1111/jrh.12394 PMC7065935

[41] Eger RJ III . Policy instruments in injury crashes: traffic law enforcement and alcohol prohibition. Transp Res Rec. 2006;1:45–9.

[42] Erickson DJ , Farbakhsh K , Toomey TL , Lenk KM , Jones‐Webb R , Nelson TF . Enforcement of alcohol‐impaired driving laws in the United States: a national survey of state and local agencies. Traffic Inj Prev. 2015;16:533–9. 25802970 10.1080/15389588.2014.995789 PMC4429770

[43] Erickson DJ , Lenk KM , Sanem JR , Nelson TF , Jones‐Webb R , Toomey TL . Current use of underage alcohol compliance checks by enforcement agencies in the United States. Alcohol Clin Exp Res. 2014;38:1712–9. 24716443 10.1111/acer.12397 PMC4062386

[44] Erickson DJ , Rutledge PC , Lenk KM , Nelson TF , Jones‐Webb R , Toomey TL . Patterns of alcohol policy enforcement activities among local law enforcement agencies: a latent class analysis. Int J Alcohol Drug Res. 2015;4:103–11. 26877822 10.7895/ijadr.v4i2.204 PMC4749155

[45] Jones‐Webb R , Toomey TL , Lenk KM , Nelson TF , Erickson DJ . Targeting adults who provide alcohol to underage youth: results from a national survey of local law enforcement agencies. J Community Health. 2015;40:569–75. 25466432 10.1007/s10900-014-9973-0 PMC4427546

[46] de Vocht F , Heron J , Angus C , Brennan A , Mooney J , Lock K , et al. Measurable effects of local alcohol licensing policies on population health in England. J Epidemiol Community Health. 2016;70:231–7. 26555369 10.1136/jech-2015-206040 PMC4789824

[47] Dula CS , Dwyer WO , LeVerne G . Policing the drunk driver: measuring law enforcement involvement in reducing alcohol‐impaired driving. J Saf Res. 2007;38:267–72. 10.1016/j.jsr.2006.10.007 17617235

[48] Eichelberger AH , McCartt AT . Impaired driving enforcement practices among state and local law enforcement agencies in the United States. J Saf Res. 2016;58:41–7. 10.1016/j.jsr.2016.06.003 27620933

[49] Fell JC , Ferguson SA , Williams AF , Fieldset M . Why are sobriety checkpoints not widely adopted as an enforcement strategy in the United States? Accid Anal Prev. 2003;35:897–902. 12971924 10.1016/s0001-4575(02)00097-0

[50] Findlay RA , Sheehan MC , Davey J , Brodie H , Rynne F . Liquor law enforcement: policy and practice in Australia. Drugs Educ Prev Policy. 2002;9:85–94.

[51] Jia K , King FJJ , Sheehan M , Ma W , Lei J , Zhang J . Drunk driving offenders' knowledge and behaviour in relation to alcohol‐involved driving in Yinchuan and a comparison with Guangzhou, China. Transp Res Part F Traffic Psychol Behav. 2016;38:182–93.

[52] Lenk KM , Nelson TF , Toomey TL , Jones‐Webb R , Erickson DJ . Sobriety checkpoint and open container laws in the United States: associations with reported drinking‐driving. Traffic Inj Prev. 2016;17:782–7. 26983365 10.1080/15389588.2016.1161759 PMC5584594

[53] Maclennan B , Kypri K , Connor J , Potiki T , Room R . New Zealand's new alcohol laws: protocol for a mixed‐methods evaluation. BMC Public Health. 2016;16:29. 26759263 10.1186/s12889-015-2638-9 PMC4710993

[54] Meesmann U , Martensen H , Dupont E . Impact of alcohol checks and social norm on driving under the influence of alcohol (DUI). Accid Anal Prev. 2015;80:251–61. 25957934 10.1016/j.aap.2015.04.016

[55] Morrison CN , Ferris J , Wiebe DJ , Peek‐Asa C , Branas CC . Sobriety checkpoints and alcohol‐involved motor vehicle crashes at different temporal scales. Am J Prev Med. 2019;56:795–802. 31005468 10.1016/j.amepre.2019.01.015 PMC6557160

[56] Randerson S , Casswell S , Huckle T . Changes in New Zealand's alcohol environment following implementation of the sale and supply of alcohol act (2012). N Z Med J. 2018;131:14–23. 29879723

[57] Sanem JR , Erickson DJ , Rutledge PC , Lenk KM , Nelson TF , Jones‐Webb R , et al. Association between alcohol‐impaired driving enforcement‐related strategies and alcohol‐impaired driving. Accid Anal Prev. 2015;78:104–9. 25756846 10.1016/j.aap.2015.02.018 PMC4390543

[58] Stringer RJ . Policing the drunk driving problem: a longitudinal examination of DUI enforcement and alcohol related crashes in the U.S. (1985–2015). Am J Crim Justice. 2019;44:474–98.

[59] Beck KH , Yan AF , Fell JC . A comparison of drivers with high versus low perceived risk of being caught and arrested for driving under the influence of alcohol. Traffic Inj Prev. 2009;10:312–9. 19593706 10.1080/15389580903022507

[60] Jia K , Fleiter J , King M , Sheehan M , Ma W , Lei J , et al. Alcohol‐related driving in China: countermeasure implications of research conducted in two cities. Accid Anal Prev. 2016;95:343–9. 26850753 10.1016/j.aap.2016.01.005

[61] Alonso F , Pastor JC , Montoro L , Esteban C . Driving under the influence of alcohol: frequency, reasons, perceived risk and punishment. Subst Abuse Treat Prev Policy. 2015;10:11. 25880078 10.1186/s13011-015-0007-4 PMC4359384

[62] Bachani AM , Risko CB , Gnim C , Coelho S , Hyder AA . Knowledge, attitudes, and practices around drinking and driving in Cambodia: 2010‐2012. Public Health. 2017;144:S32–8. 10.1016/j.puhe.2016.12.012 28288729

[63] Casswell S , Meier P , MacKintosh AM , Brown A , Hastings G , Thamarangsi T , et al. The international alcohol control (IAC) study‐evaluating the impact of alcohol policies. Alcohol Clin Exp Res. 2012;36:1462–7. 22404733 10.1111/j.1530-0277.2012.01738.x

[64] Dent CW , Grube JW , Biglan A . Community level alcohol availability and enforcement of possession laws as predictors of youth drinking. Prev Med. 2005;40:355–62. 15533551 10.1016/j.ypmed.2004.06.014 PMC3707296

[65] Lipperman‐Kreda S , Grube JW , Paschall MJ . Community norms, enforcement of minimum legal drinking age laws, personal beliefs and underage drinking: an explanatory model. J Community Health. 2010;35:249–57. 20135210 10.1007/s10900-010-9229-6 PMC2863071

[66] Lipperman‐Kreda S , Paschall M‐J , Grube JW . Perceived local enforcement, personal beliefs, and underage drinking: an assessment of moderating and main effects. J Stud Alcohol Drugs. 2009;70:64–9. 19118393 10.15288/jsad.2009.70.64 PMC2629628

