# Supplementary material for: Quantitative tools and measurements for assessing the implementation of regulatory policies in reducing alcohol consumption and alcohol‐related harms: A scoping review
Source: Drug Alcohol Rev. 2022 Sep 12;42(1):157–68. doi: 10.1111/dar.13543 (PMC10087297; doi:10.1111/dar.13543)
Supplement: Supplementary file 1 — Table S1 Search terms across different databases [file DAR-42-157-s002.docx]

**Supporting Information**

**Table S1**. Search terms across different databases

| **Search terms** | **Number of studies found** |
| --- | --- |
| Scopus |  |
| ( ( TITLE-ABS ( alcohol* W/10 polic* ) ) AND ( TITLE-ABS-KEY( implement* OR enforc* OR monitor* OR compliance ))) OR ( ( TITLE-ABS ( alcohol* W/10 regulat* ) ) AND ( TITLE-ABS-KEY( implement* OR enforc* OR monitor* OR compliance ) )) OR ( ( TITLE-ABS ( alcohol* W/10 strateg* ) ) AND ( TITLE-ABS-KEY( implement* OR enforc* OR monitor* OR compliance ) )) OR ( ( TITLE-ABS ( alcohol* W/10 law* ) ) AND ( TITLE-ABS-KEY(implement* OR enforc* OR monitor* OR compliance ) )) OR ( ( TITLE-ABS ( alcohol* W/10 legislation ) ) AND ( TITLE-ABS-KEY( implement* OR enforc* OR monitor* OR compliance ))) AND NOT (TITLE-ABS ("non-alcohol*" OR “brief screening” OR screening OR “alcohol treatment” )) AND NOT SUBJAREA ( vete ) AND ( LIMIT-TO ( PUBYEAR , 2021 ) OR LIMIT-TO ( PUBYEAR , 2020 ) OR LIMIT-TO ( PUBYEAR , 2019 ) OR LIMIT-TO ( PUBYEAR , 2018 ) OR LIMIT-TO ( PUBYEAR , 2017 ) OR LIMIT-TO ( PUBYEAR , 2016 ) OR LIMIT-TO ( PUBYEAR , 2015 ) OR LIMIT-TO ( PUBYEAR , 2014 ) OR LIMIT-TO ( PUBYEAR , 2013 ) OR LIMIT-TO ( PUBYEAR , 2012 ) OR LIMIT-TO ( PUBYEAR , 2011 ) OR LIMIT-TO ( PUBYEAR , 2010 ) OR LIMIT-TO ( PUBYEAR , 2009 ) OR LIMIT-TO ( PUBYEAR , 2008 ) OR LIMIT-TO ( PUBYEAR , 2007 ) OR LIMIT-TO ( PUBYEAR , 2006 ) OR LIMIT-TO ( PUBYEAR , 2005 ) OR LIMIT-TO ( PUBYEAR , 2004 ) OR LIMIT-TO ( PUBYEAR , 2003 ) OR LIMIT-TO ( PUBYEAR , 2002 ) OR LIMIT-TO ( PUBYEAR , 2001 ) OR LIMIT-TO ( PUBYEAR , 2000 ) ) AND ( LIMIT-TO ( DOCTYPE , "ar" ) OR LIMIT-TO ( DOCTYPE , "re" ) OR LIMIT-TO ( DOCTYPE , "cp" ) OR LIMIT-TO ( DOCTYPE , "sh" ) ) AND ( LIMIT-TO ( LANGUAGE , "English" ) ) | 1,904 |
| ( TITLE-ABS ( taxes OR tax OR taxation OR pric* OR affordability OR "minimum pricing" OR "minimum unit pricing" OR "floor price" ) ) AND ( TITLE-ABS ( alcohol* ) ) AND (TITLE-ABS-KEY( implement* OR enforc* OR monitor* OR compliance)) AND NOT "non-alcohol*" AND NOT SUBJAREA ( vete ) AND ( LIMIT-TO ( PUBYEAR , 2021 ) OR LIMIT-TO ( PUBYEAR , 2020 ) OR LIMIT-TO ( PUBYEAR , 2019 ) OR LIMIT-TO ( PUBYEAR , 2018 ) OR LIMIT-TO ( PUBYEAR , 2017 ) OR LIMIT-TO ( PUBYEAR , 2016 ) OR LIMIT-TO ( PUBYEAR , 2015 ) OR LIMIT-TO ( PUBYEAR , 2014 ) OR LIMIT-TO ( PUBYEAR , 2013 ) OR LIMIT-TO ( PUBYEAR , 2012 ) OR LIMIT-TO ( PUBYEAR , 2011 ) OR LIMIT-TO ( PUBYEAR , 2010 ) OR LIMIT-TO ( PUBYEAR , 2009 ) OR LIMIT-TO ( PUBYEAR , 2008 ) OR LIMIT-TO ( PUBYEAR , 2007 ) OR LIMIT-TO ( PUBYEAR , 2006 ) OR LIMIT-TO ( PUBYEAR , 2005 ) OR LIMIT-TO ( PUBYEAR , 2004 ) OR LIMIT-TO ( PUBYEAR , 2003 ) OR LIMIT-TO ( PUBYEAR , 2002 ) OR LIMIT-TO ( PUBYEAR , 2001 ) OR LIMIT-TO ( PUBYEAR , 2000 ) ) AND ( LIMIT-TO ( DOCTYPE , "ar" ) OR LIMIT-TO ( DOCTYPE , "re" ) OR LIMIT-TO ( DOCTYPE , "cp" ) OR LIMIT-TO ( DOCTYPE , "bz" ) OR LIMIT-TO ( DOCTYPE , "sh" ) ) AND ( LIMIT-TO ( LANGUAGE , "English" ) ) | 407 |
| ( TITLE-ABS (sale* OR “trading hour*” OR “intoxicat*” OR "on-premis*" OR "off-premis*" OR "minimum age" OR "minimum legal age" OR availabilit* OR retail* OR outlet* OR licen* OR "online deliver*" OR "online sale*" ) ) AND ( TITLE-ABS ( alcohol* ) ) AND (TITLE-ABS-KEY( implement* OR enforc* OR monitor* OR compliance )) AND NOT "non-alcohol*" AND NOT SUBJAREA ( vete ) AND ( LIMIT-TO ( PUBYEAR , 2021 ) OR LIMIT-TO ( PUBYEAR , 2020 ) OR LIMIT-TO ( PUBYEAR , 2019 ) OR LIMIT-TO ( PUBYEAR , 2018 ) OR LIMIT-TO ( PUBYEAR , 2017 ) OR LIMIT-TO ( PUBYEAR , 2016 ) OR LIMIT-TO ( PUBYEAR , 2015 ) OR LIMIT-TO ( PUBYEAR , 2014 ) OR LIMIT-TO ( PUBYEAR , 2013 ) OR LIMIT-TO ( PUBYEAR , 2012 ) OR LIMIT-TO ( PUBYEAR , 2011 ) OR LIMIT-TO ( PUBYEAR , 2010 ) OR LIMIT-TO ( PUBYEAR , 2009 ) OR LIMIT-TO ( PUBYEAR , 2008 ) OR LIMIT-TO ( PUBYEAR , 2007 ) OR LIMIT-TO ( PUBYEAR , 2006 ) OR LIMIT-TO ( PUBYEAR , 2005 ) OR LIMIT-TO ( PUBYEAR , 2004 ) OR LIMIT-TO ( PUBYEAR , 2003 ) OR LIMIT-TO ( PUBYEAR , 2002 ) OR LIMIT-TO ( PUBYEAR , 2001 ) OR LIMIT-TO ( PUBYEAR , 2000 ) ) AND ( LIMIT-TO ( DOCTYPE , "ar" ) OR LIMIT-TO ( DOCTYPE , "re" ) OR LIMIT-TO ( DOCTYPE , "cp" ) OR LIMIT-TO ( DOCTYPE , "sh" ) OR LIMIT-TO ( DOCTYPE , "bz" ) ) AND ( LIMIT-TO ( LANGUAGE , "English" ) ) | 2,189 |
| ( TITLE-ABS ( marketing OR promot* OR adverti* OR sponsor* ) ) AND ( TITLE-ABS ( alcohol* ) ) AND (TITLE-ABS-KEY( implement* OR enforc* OR monitor* OR compliance )) AND NOT "non-alcohol*" AND NOT SUBJAREA ( vete ) AND ( LIMIT-TO ( PUBYEAR , 2021 ) OR LIMIT-TO ( PUBYEAR , 2020 ) OR LIMIT-TO ( PUBYEAR , 2019 ) OR LIMIT-TO ( PUBYEAR , 2018 ) OR LIMIT-TO ( PUBYEAR , 2017 ) OR LIMIT-TO ( PUBYEAR , 2016 ) OR LIMIT-TO ( PUBYEAR , 2015 ) OR LIMIT-TO ( PUBYEAR , 2014 ) OR LIMIT-TO ( PUBYEAR , 2013 ) OR LIMIT-TO ( PUBYEAR , 2012 ) OR LIMIT-TO ( PUBYEAR , 2011 ) OR LIMIT-TO ( PUBYEAR , 2010 ) OR LIMIT-TO ( PUBYEAR , 2009 ) OR LIMIT-TO ( PUBYEAR , 2008 ) OR LIMIT-TO ( PUBYEAR , 2007 ) OR LIMIT-TO ( PUBYEAR , 2006 ) OR LIMIT-TO ( PUBYEAR , 2005 ) OR LIMIT-TO ( PUBYEAR , 2004 ) OR LIMIT-TO ( PUBYEAR , 2003 ) OR LIMIT-TO ( PUBYEAR , 2002 ) OR LIMIT-TO ( PUBYEAR , 2001 ) OR LIMIT-TO ( PUBYEAR , 2000 ) ) AND ( LIMIT-TO ( DOCTYPE , "ar" ) OR LIMIT-TO ( DOCTYPE , "re" ) OR LIMIT-TO ( DOCTYPE , "cp" ) OR LIMIT-TO ( DOCTYPE , "sh" ) OR LIMIT-TO ( DOCTYPE , "bz" ) ) AND ( LIMIT-TO ( LANGUAGE , "English" ) ) | 1,982 |
| ( TITLE-ABS ( driv* OR “blood alcohol concentration” ) ) AND ( TITLE-ABS ( alcohol* ) ) AND (TITLE-ABS-KEY ( implement* OR enforc* OR monitor* OR compliance)) AND NOT "non-alcohol*" AND NOT SUBJAREA ( vete ) AND ( LIMIT-TO ( PUBYEAR , 2021 ) OR LIMIT-TO ( PUBYEAR , 2020 ) OR LIMIT-TO ( PUBYEAR , 2019 ) OR LIMIT-TO ( PUBYEAR , 2018 ) OR LIMIT-TO ( PUBYEAR , 2017 ) OR LIMIT-TO ( PUBYEAR , 2016 ) OR LIMIT-TO ( PUBYEAR , 2015 ) OR LIMIT-TO ( PUBYEAR , 2014 ) OR LIMIT-TO ( PUBYEAR , 2013 ) OR LIMIT-TO ( PUBYEAR , 2012 ) OR LIMIT-TO ( PUBYEAR , 2011 ) OR LIMIT-TO ( PUBYEAR , 2010 ) OR LIMIT-TO ( PUBYEAR , 2009 ) OR LIMIT-TO ( PUBYEAR , 2008 ) OR LIMIT-TO ( PUBYEAR , 2007 ) OR LIMIT-TO ( PUBYEAR , 2006 ) OR LIMIT-TO ( PUBYEAR , 2005 ) OR LIMIT-TO ( PUBYEAR , 2004 ) OR LIMIT-TO ( PUBYEAR , 2003 ) OR LIMIT-TO ( PUBYEAR , 2002 ) OR LIMIT-TO ( PUBYEAR , 2001 ) OR LIMIT-TO ( PUBYEAR , 2000 ) ) AND ( LIMIT-TO ( DOCTYPE , "ar" ) OR LIMIT-TO ( DOCTYPE , "re" ) OR LIMIT-TO ( DOCTYPE , "cp" ) OR LIMIT-TO ( DOCTYPE , "sh" ) ) AND ( LIMIT-TO ( LANGUAGE , "English" ) ) | 1,778 |
| ( ( ( TITLE-ABS ( alcohol* W/10 polic* ) ) AND ( TITLE-ABS-KEY ( implement* OR enforc* OR monitor* OR compliance ) ) ) OR ( ( TITLE-ABS ( alcohol* W/10 regulat* ) ) AND ( TITLE-ABS-KEY ( implement* OR enforc* OR monitor* OR compliance ) ) ) OR ( ( TITLE-ABS ( alcohol* W/10 strateg* ) ) AND ( TITLE-ABS-KEY ( implement* OR enforc* OR monitor* OR compliance ) ) ) OR ( ( TITLE-ABS ( alcohol* W/10 law* ) ) AND ( TITLE-ABS-KEY ( implement* OR enforc* OR monitor* OR compliance ) ) ) OR ( ( TITLE-ABS ( alcohol* W/10 legislation ) ) AND ( TITLE-ABS-KEY ( implement* OR enforc* OR monitor* OR compliance ) ) ) AND NOT ( TITLE-ABS ( "non-alcohol*" OR "brief screening" OR screening OR "alcohol treatment" ) ) AND NOT SUBJAREA ( vete ) ) OR ( ( TITLE-ABS ( taxes OR tax OR taxation OR pric* OR affordability OR "minimum pricing" OR "minimum unit pricing" OR "floor price" ) ) AND ( TITLE-ABS ( alcohol* ) ) AND ( TITLE-ABS-KEY ( implement* OR enforc* OR monitor* OR compliance ) ) AND NOT "non-alcohol*" AND NOT SUBJAREA ( vete ) ) OR ( ( TITLE-ABS ( sale* OR "trading hour*" OR "intoxicat*" OR "on-premis*" OR "off-premis*" OR "minimum age" OR "minimum legal age" OR availabilit* OR retail* OR outlet* OR licen* OR "online deliver*" OR "online sale*" ) ) AND ( TITLE-ABS ( alcohol* ) ) AND ( TITLE-ABS-KEY ( implement* OR enforc* OR monitor* OR compliance ) ) AND NOT "non-alcohol*" AND NOT SUBJAREA ( vete ) ) OR ( ( TITLE-ABS ( marketing OR promot* OR adverti* OR sponsor* ) ) AND ( TITLE-ABS ( alcohol* ) ) AND ( TITLE-ABS-KEY ( implement* OR enforc* OR monitor* OR compliance ) ) AND NOT "non-alcohol*" AND NOT SUBJAREA ( vete ) ) OR ( ( TITLE-ABS ( driv* OR "blood alcohol concentration" ) ) AND ( TITLE-ABS ( alcohol* ) ) AND ( TITLE-ABS-KEY ( implement* OR enforc* OR monitor* OR compliance ) ) AND NOT "non-alcohol*" AND NOT SUBJAREA ( vete ) ) AND ( LIMIT-TO ( LANGUAGE , "English" ) ) AND ( LIMIT-TO ( DOCTYPE , "ar" ) OR LIMIT-TO ( DOCTYPE , "re" ) OR LIMIT-TO ( DOCTYPE , "cp" ) OR LIMIT-TO ( DOCTYPE , "sh" ) OR LIMIT-TO ( DOCTYPE , "bz" ) ) AND ( LIMIT-TO ( PUBYEAR , 2021 ) OR LIMIT-TO ( PUBYEAR , 2020 ) OR LIMIT-TO ( PUBYEAR , 2019 ) OR LIMIT-TO ( PUBYEAR , 2018 ) OR LIMIT-TO ( PUBYEAR , 2017 ) OR LIMIT-TO ( PUBYEAR , 2016 ) OR LIMIT-TO ( PUBYEAR , 2015 ) OR LIMIT-TO ( PUBYEAR , 2014 ) OR LIMIT-TO ( PUBYEAR , 2013 ) OR LIMIT-TO ( PUBYEAR , 2012 ) OR LIMIT-TO ( PUBYEAR , 2011 ) OR LIMIT-TO ( PUBYEAR , 2010 ) OR LIMIT-TO ( PUBYEAR , 2009 ) OR LIMIT-TO ( PUBYEAR , 2008 ) OR LIMIT-TO ( PUBYEAR , 2007 ) OR LIMIT-TO ( PUBYEAR , 2006 ) OR LIMIT-TO ( PUBYEAR , 2005 ) OR LIMIT-TO ( PUBYEAR , 2004 ) OR LIMIT-TO ( PUBYEAR , 2003 ) OR LIMIT-TO ( PUBYEAR , 2002 ) OR LIMIT-TO ( PUBYEAR , 2001 ) OR LIMIT-TO ( PUBYEAR , 2000 ) ) | 6,102 |
| Web of Science |  |
| ((AB=(alcohol* NEAR polic*) AND AB=(implement* OR enforc* OR monitor* OR compliance)) OR (AB=(alcohol* NEAR regulat*) AND AB=(implement* OR enforc* OR monitor* OR compliance)) OR (AB=(alcohol* NEAR strateg*) AND AB=(implement* OR enforc* OR monitor* OR compliance)) OR (AB=(alcohol* NEAR law*) AND AB=(implement* OR enforc* OR monitor* OR compliance)) OR (AB=(alcohol* NEAR legislation) AND AB=(implement* OR enforc* OR monitor* OR compliance))) NOT (AB=("non-alcohol*" OR “brief screening” OR screening OR “alcohol treatment”)) NOT SU= Zoology | 2,656 |
| AB=(taxes OR tax OR taxation OR pric* OR affordability OR "minimum pricing" OR "minimum unit pricing" OR "floor price") AND AB=( alcohol* ) AND AB=( implement* OR enforc* OR monitor* OR compliance ) NOT AB="non-alcohol*" NOT SU= Zoology | 567 |
| AB=( sale* OR “trading hour*” OR “intoxicat*” OR "on-premis*" OR "off-premis*" OR "minimum age" OR "minimum legal age" OR availabilit* OR retail* OR outlet* OR licen* OR "online deliver*" OR "online sale*") AND AB=( alcohol* ) AND AB=( implement* OR enforc* OR monitor* OR compliance ) NOT AB= “non-alcohol*” NOT SU= Zoology | 2,410 |
| AB=( marketing OR promot* OR adverti* OR sponsor*) AND AB=( alcohol*) AND AB=( implement* OR enforc* OR monitor* OR compliance) NOT AB="non-alcohol*" NOT SU= Zoology | 2,687 |
| AB=( driv* OR “blood alcohol concentration” ) AND AB=( alcohol* ) AND AB=( implement* OR enforc* OR monitor* OR compliance ) NOT AB= "non-alcohol*" NOT SU= Zoology | 1,889 |
| Combined with all  #5 OR #4 OR #3 OR #2 OR #1  Refined by: PUBLICATION YEARS: ( 2021 OR 2005 OR 2020 OR 2004 OR 2019 OR 2003 OR 2018 OR 2002 OR 2017 OR 2001 OR 2016 OR 2000 OR 2015 OR 2014 OR 2013 OR 2012 OR 2011 OR 2010 OR 2009 OR 2008 OR 2007 OR 2006 ) AND DOCUMENT TYPES: ( ARTICLE OR REPORT OR REVIEW OR THESIS DISSERTATION OR CASE REPORT OR CLINICAL TRIAL ) AND LANGUAGES: ( ENGLISH ) | 5,549 |
