# Supplementary material for: Quantitative tools and measurements for assessing the implementation of regulatory policies in reducing alcohol consumption and alcohol‐related harms: A scoping review
Source: Drug Alcohol Rev. 2022 Sep 12;42(1):157–68. doi: 10.1111/dar.13543 (PMC10087297; doi:10.1111/dar.13543)
Supplement: Supplementary file 3 — Table S3 Summary of included tools and measurements for assessing policy implementation across four policies [file DAR-42-157-s001.docx]

**Table S3**: Summary of included tools and measurements for assessing policy implementation across four policies

| **tim** | **Author** | **Year** | **Title** | **Aims** | **Country of origin and level of the study** | **Population** | **Policy areas** | **Methods** | **Tools used** | **Measurements** | **Validity and reliability** |
| --- | --- | --- | --- | --- | --- | --- | --- | --- | --- | --- | --- |
| Physical availability control | | | | | | | | | | | |
| 1. | De Vocht F, Heron J, Angus C, Brennan A, Mooney J, Lock K, et al. | 2016 | Measurable effects of local alcohol licensing policies on population health in England | To investigate the impacts of intensity of enforcement of CIZ in England | England and Wales, national level | Alcohol retailers | Physical availability: licensing | **Study design and data collection methods**: The study used secondary data from two main sources. First, level of enforcement of CIZ, the study used data from the Home Office’s Alcohol and Late-Night Refreshment Licensing England and Wales. Second, the hospital admission was based on data specified as alcohol-related problems, based on diagnosis.  **Data analysis method:** Hierarchical growth model | Secondary sources | **Main independent variables:** intensity of enforcement of CIZ.  The intensity of enforcement was estimated by two different measures: (i) the authors measured whether CIZ was used (yes/no); (ii) whether any licenses for new premises were successfully challenged by the lower tier local authorities in a particular year (yes/no).  Then, it was coded into three categories: (i) no CIZ used and no licensing applications refused; (ii) applied either one of the measures; (iii) applied both measures.  **Main dependent variables:** aged standardised alcohol-related hospital per 100,000 population.  **Adjusted variables:** deprivation index, numbers of population, alcohol-related crime control to control for non-random implementation of policies in lower tier local authorities (e.g., more intense alcohol policies were more likely to be implemented in areas with more problems). | None* |
| 2 | Dent CW, Grube JW & Biglan A | 2005 | Community level alcohol availability and enforcement of possession laws as predictors of youth drinking | To assess relationship between commercial access to alcohol, the enforcement of possession laws and alcohol use among youth | USA, state level, Oregon | Community and youth grade 11 | Physical availability: commercial access and minimum purchasing age | **Study design and data collection methods:** The study used a cross-sectional survey. Researchers randomly selected communities and schools in the catchment area. The study collected data from students in grades 8 and 11 each year, but in different cohorts. However, the study used data from students in grade 11 collected in 2001.  **Data analysis methods:** Multilevel analysis. | Used questionnaires | **Main independent variables:** perception on law enforcement (likelihood of being caught).  **Main dependent variables:** alcohol use, binge drinking, drink in school and drink-driving.  **Adjusted variables:** sources of alcohol (e.g., from friends, parents or any social place) and the commercial source (e.g., grocery stores, convenience stores, drug stores and gas stations). | None |
| 3 | Erickson DJ, Lenk KM, Sanem JR, Nelson TF, Jones-Webb R & Toomey TL | 2014 | Current use of underage alcohol compliance checks by enforcement agencies in the United States | To assess numbers of agencies conducted compliance checks, and numbers of agencies that conducted compliance check with optimal methods and characteristics of those agencies | USA, national level | Law enforcement agencies | Physical availability: minimum purchasing age | **Study design and data collection methods:** The study employed a cross-sectional survey and applied stratified sampling methods to select enforcement agencies and state agencies. Staff who were knowledgeable about law enforcement were interviewed by phone or completed questionnaires online.  **Data analysis methods:** Multiple logistic regression | Used questionnaires | **Main independent variables:** (i) number of full-time officers per 1,000 population; (ii) numbers of full-time staff that are responsible for law enforcement; (iii) having an alcohol-related division.  **Main dependent variables:** law enforcement agencies that conducted compliance checks (yes/no/do not know), and frequency of compliance checks.  **Adjusted variables:** community level: population size, percent of ethnicity (i.e., Black and Hispanic), percent living in poverty, percent aged 15-20 years. | None |
| 4 | Erickson DJ, Lenk KM, Toomey TL, Nelson TF & Jones-Webb R | 2016 | The alcohol policy environment, enforcement and consumption in the United States | To assess alcohol policy environment, enforcement and alcohol consumption | USA, national level | Local enforcement agencies | Physical availability control: underage alcohol use (e.g., underage possession, consumption, internal possession, purchasing, false ID users and use/lose driving privileges), provision of alcohol to underage (e.g., keg registration, underage furnishing, hosting underage drinking parties, false ID suppliers), alcohol serving (e.g., age of servers, beverage service training), general availability | **Study design and data collection methods:** The study used data from various sources: (i) strength of policy collected from the Alcohol Policy Information System database; (ii) conducting a cross-sectional survey among enforcement agencies using multi-stage sampling; (iii) consumption measures and individual-level demographic measures were gathered from the Behavioral Risk Factor Surveillance System; (iv) state-level demographic measures obtained from secondary data sources.  **Analysis methods:** Multilevel logistic regression. | Used questionnaires and secondary data sources | **Main independent variables:** (i) restrictiveness of alcohol policy at state level; (ii) presence and absence of enforcement activities and its intensity (i.e., frequency of compliance checks and coverage of alcohol outlets). The study created as a latent class and group into low, moderate, high class for enforcement.  **Main dependent variables:** alcohol use during past month, binge drinking and heavy drinking.  **Adjusted variables:** individual characteristics (i.e., sex, age, marital status and education) and state demographic profiles (i.e., population and unemployment rate, and religiosity) | None |
| 5 | Findlay RA, Sheehan MC, Davey J, Brodie H & Rynne F | 2002 | Liquor law enforcement: policy and practice in Australia | To examine inadequacies in liquor-licensing law enforcement in Australia | Australia, sub-national level, in Queensland | Police officers | Physical availability: access to alcohol among youths, restricting trading hours, supplying alcohol to intoxicated persons | **Study design and data collection methods:**  The study employed a cross-sectional survey by sending questionnaires to police officers in urban and rural areas.  **Data analysis:** Used chi-square tests to compare enforcement outcomes between urban and rural areas. | Used questionnaires | **Main independent variables:** self-report on the knowledge of alcohol control law (using a Likert scale ranging from ‘very good’ to ‘very poor’), urban and rural areas.  **Main dependent variables:** enforcement activities (yes/no), and percentage of respondents who breached the alcohol control law (vendors or individuals).  **Adjusted variables:** none. | Content validity |
| 6 | Jones-Webb R, Toomey TL, Lenk KM, Nelson TF & Erickson DJ | 2015 | Targeting adults who provide alcohol to underage youth: results from a national survey of local law enforcement agencies | To investigate the factors that influence law enforcement agencies to conduct enforcement activities in USA | USA, national level | Law enforcement agencies | Physical activities: social supply of alcohol to underage youth | **Study design and data collection methods:** Cross-sectional survey. A multistage strategy was used to select local law enforcement agencies. The states were divided into large and small. For large states, 40 agencies were selected and 20 agencies for small states. At each agency, the agents or officers who were most knowledgeable about the agency’s enforcement activities, were invited to join the survey. The survey was conducted by telephone interview and self-administered via an online platform.  **Data analysis methods:** Multiple logistic regression | Used questionnaires | **Main independent variables:** (i) number of full-time officers specific to alcohol enforcement per 1,000 population; and (ii) division specific to alcohol enforcement.  **Main dependent variables:** conducting law enforcement (yes/no).  **Adjusted variables:** Local level: commonness of underage drinking, regional level (wet, moderate and dry areas), total population, proportion of African American/Black, proportion of Hispanic, proportion of population below poverty line and proportion aged between 15-20 years. | None |
| 7 | Lipperman-Kreda S, Grube JW & Paschall MJ | 2010 | Community norms, enforcement of minimum legal drinking age laws, personal beliefs and underage drinking: An explanatory model | To investigate associations between local policy and enforcement, community norms, and personal beliefs | USA, state level | Students at grade 8th and 11th | Physical availability: minimum drinking age | **Study design and data collection methods:** Cross-sectional survey among students in grade 8 and 11. A self-administered questionnaire was used and supervised by teachers.  **Data analysis methods:** Structural equations analysis. | Used questionnaires | **Main independent variables:** community’s disapproval of alcohol use (Likert scale), perceived local alcohol policy and enforcement (Likert scales), and personal beliefs on alcohol use (Likert scales), perceived alcohol availability (Likert scales), perceived alcohol-related harm (Likert scale) and number of friends who consumed alcohol.  **Main dependent variables:** adolescents’ alcohol use in the past 30 days.  **Adjusted variables:** gender, age and ethnicity. | None |
| 8 | Lipperman-Kreda S, Paschall MJ & Grube JW | 2009 | Perceived local enforcement, personal beliefs and underage drinking: An assessment of moderating and main effects | To investigate relationships between perceived enforcement of underage drinking laws and personal beliefs and underage drinking | USA, sub-national conducted in Oregon | Students, grade 8th and 11th | Physical availability: minimum drinking age | **Study design and data collection methods:** Cross-sectional survey design using a self-administered questionnaire.  **Data analysis methods:** Linear regression. | Used questionnaires | **Main independent variables:** perceived of alcohol enforcement (Likert scales, likelihood of being caught).  **Main dependent variables:** underage drinking.  **Adjusted variables:** The model was adjusted by individual characteristics: gender, ethnicity, age, alcohol use before the age of 12 years and personal beliefs (i.e., perceived alcohol availability) (Likert scales), perceived alcohol-related harm (Likert scales) and personal disapproval of alcohol use (Likert scales). | None |
| Drink-driving policy | | | | | | | | | | | |
| 9 | Alonso F, Pastor JC, Montoro L & Esteban C | 2015 | Driving under the influence of alcohol: frequency, reasons, perceived risk and punishment | To investigate frequency, reasons, and perceived risk and punishment of drink driving behaviours | Spain, national level | General population | Drink driving measures | **Study design and data collection methods:** Cross-sectional study, using telephone interview. The subjects were randomly selected using random digit dialling. One member who had a driving license was randomly selected.  **Data analysis methods:** Student t-test and one-way ANOVA. | Used questionnaires | **Main independent variables:** (i) reason for (not) drink-driving; (ii) perceived risk of being caught for drink-driving (Likert scales); (iii) perception toward punishment (Likert scales); (iv) experiences of punishment.  **Main dependent variables:** frequency of drink driving.  **Adjusted variables:** age and gender. | None |
| 10 | Bachani AM, Risko CB, Gnim C, Coelho S & Hyder AA | 2017 | Knowledge, attitudes and practices around drinking and driving in Cambodia: 2010-2012 | To illustrate current knowledge, attitudes and practices around drink-driving in three Cambodian provinces | Cambodia, national level | Road users, aged 18 years old and above | Drink-driving measures | **Study design and data collection methods:** Cross-sectional survey, roadside survey in three provinces in Cambodia. Multiphase sampling was used. Roads with gas stations were selected and included all types of roads (highway, secondary roads, city roads and rural roads). Road users were randomly selected and interviewed.  **Data analysis methods:** Chi-square test. | Used questionnaires | **Main independent variables:** (i) drivers’ attitudes toward drink-driving; (ii) perceived risks of drink-driving; (iii) awareness of social media related to risk factors and enforcement; (iv) police enforcement levels.  **Main dependent variables:** drink-driving behaviours.  **Adjusted variables:** None. The study measured demographic characteristics (age, sex, education) but did not adjust in the analysis. | None |
| 11 | Beck KH, Fell JC & Yan AF | 2009 | A comparison of drivers with high versus low perceived risk of being caught and arrested for driving under the influence of alcohol | To compare perceived risk of being caught, high vs low on drink driving behaviours | USA, state level | General population | Drink driving measures | **Study design and data collection methods:** Cross-sectional survey among law enforcement officers using telephone interviews. The participants were randomly selected from a random-digit-dial telephone survey.  **Data analysis methods:** Logistic regression. | Used questionnaires | **Main independent variables:** belief about effectiveness of sobriety checkpoints, traffic citation history, and knowledge about drink-driving laws (correct/incorrect responses).  **Main dependent variables:** perceived likelihood of being arrested or stopped by the police (Likert scale).  **Adjusted variables:** age, gender, race, frequency of automobile use and household income. | None |
| 12 | Dula CS, Dwyer WO & LeVerne G | 2007 | Policing the drunk driver: measuring law enforcement involvement in reducing alcohol-impaired driving | To investigate relationship between and DUI arrests and DUI-related crashes | USA, state level | General population | Drink driving measures | **Study design and data collection methods:** The study used secondary data.  **Data analysis methods:** Pearson correlation coefficient. | Secondary data | **Main independent variables:** DUI arrest.  **Main dependent variables:** DUI-related crashes.  **Adjusted variables:** numbers of driving license. | None |
| 13 | Eger RJ, III | 2006 | Policy instruments in injury crashes: traffic law enforcement and alcohol prohibition | To investigate association between law enforcement, alcohol-related policies and injury crashes | USA, state level | Counties | Drink driving measures | Quantitative study, **study design and data collection methods:** The study used secondary sources from Kentucky's 120 counties.  **Data analysis methods:** Negative binomial regression. | Secondary data sources | **Main independent variables:** (i) using number of police officers and sheriff deputies as a proxy of law enforcement; (ii) alcohol policies at county level.  **Main dependent variables:** numbers of injuries from vehicle crashes.  **Adjusted variables:** number of two-lane roads, population density, number of males aged 18-24 years old, population over 65 years old, number of people driving under the influence, convictions in the county and number of suspended license drivers. | None |
| 14 | Eichelberger AH & McCartt AT | 2016 | Impaired driving enforcement practices among state and local law enforcement agencies in the United States | To investigate actual implementation of drink driving measures in the USA | USA, national level | Law enforcement agencies | Drink driving measures | S**tudy design and data collection methods:** Cross-sectional survey. A stratified sampling method was used to select enforcement agencies. The survey was conducted among enforcement agencies using telephone interviews.  **Data analysis methods:** Descriptive analysis. | Used questionnaires | **Variables of interest:** (i) types of training officers received for DUI driving enforcement; (ii) agency’s driving under influence of alcohol enforcement activity (e.g., types of enforcement activities, frequency of enforcement activities, whether activities were publicised); (iii) challenge of inaction in driving under influence of alcohol enforcement; (iv) training of law enforcement. | None |
| 15 | Erickson DJ, Farbakhsh K, Toomey TL, Lenk KM, Jones-Webb R & Nelson TF | 2015 | Enforcement of alcohol-impaired driving laws in the United States: a national survey of state and local agencies | To investigate enforcement strategies used by state and local law enforcement agencies to prevent alcohol-impaired driving law | USA, national level | Law enforcement agencies | Drink driving measures | **Study design and data collection methods:** Cross-sectional survey among enforcement agencies at state and local level. At state level, it applied random selection of the state agencies. At the local level, enforcement agencies were selected using multi-stage sampling. Telephone/online surveys were used to collect data from the most knowledgeable persons from different agencies.  **Data analysis methods:** Multiple logistic regression. | Used questionnaires | **Main independent variables:** the percentage of total annual resources in their agencies devoting for enforcing drink-driving laws, region (wet, moderate and dry), population of jurisdiction, number of full-time officers per 1,000 population, having a division specifically to enforce alcohol laws, having an officer assigned to alcohol enforcement.  **Main dependent variables:** frequency of enforcement agencies in different strategies, including sobriety checkpoints, saturation patrols, and open container law.  **Adjusted variables:** percent of Black, percentage living under the poverty line and percentage of the population aged 15-30 years, and commonness of drink-driving. | None |
| 16 | Fell JC, Ferguson SA, Williams AF & Fields M | 2003 | Why are sobriety checkpoints not widely adopted as an enforcement strategy in the United States? | To identify factors that influence efforts of conducting sobriety checkpoints. | USA, state level, 37 states and the district of Columbia | Enforcement agencies | Drink driving measures | **Study design and data collection methods:** The study applied mixed methods. A cross-sectional survey was used to examine frequency of sobriety checkpoints among state agencies that enforce drink-driving measures. Then, five infrequent and five frequent sobriety checkpoints were chosen to explore factors that influence sobriety checkpoints. An in-depth interview was applied to gather information from officers.  **Data analysis methods:** Descriptive analysis. | Used questionnaires | **Variables of interest:** frequency of conducting sobriety checkpoints, and frequency and platforms of publicity of sobriety checkpoints | None |
| 17 | Fell JC, Waehrer G, Voas RB, Auld-Owens A, Carr K & Pell K | 2014 | Effects of enforcement intensity on alcohol impaired driving crashes | To investigate enforcement intensity on the rate of crashes | USA, national level | General population | Drink driving measures | **Study design and data collection methods:** The study used a cross-sectional survey, national roadside survey. The data were collected by self-report and breath test.  **Data analysis methods:** Log-linear regression with robust standard errors. | Used questionnaires | **Main independent variables:** (i) annual number of driving under influence arrests per capita; (ii) frequency of sobriety checkpoints; (iii) annual number of traffic stops per capita; (iv) number of sworn officers per capita; (v) number of other traffic enforcement citations per capita, (e.g., seat belt citations, speeding tickets, and other moving violations and warnings); (vi) impaired drink driving rates, BAC levels ≥0.08, ≥0.05, ≥0.08.  **Main dependent variables:** alcohol-impaired driving crashes.  **Adjusted variables:** Community level: unemployment rates, the prevalence of alcohol-related night-time activities and alcohol availability in the primary sampling unit. | None |
| 18 | Fell JC, Waehrer G, Voas RB, Auld-Owens A, Carr K & Pell K | 2015 | Relationship of impaired-driving enforcement intensity to drinking and driving on the roads | To investigate associations between enforcement intensity and drunk driving behaviours | USA, national level | Enforcement agencies | Drink driving measures | **Study design and data collection methods:** The study employed multisource data. The data included three main sources: (i) The NRS employed face-to-face interview drivers who were randomly selected at 300 locations across 60 primary sampling unit; (ii) a telephone interview among police officers. The survey was undertaken among the police officers that involved in NRS; (iii) Census data.  **Data analysis methods:** Multiple logistic regression | Used questionnaires and secondary data | **Main independent variables:** six enforcement activities, including: (i) DUI of alcohol arrests; (ii) DUI saturation patrols per 10,000 population; (iii) traffic stops per 10,000 population; (iv) sworn officers per 10,000 population; (v) other enforcement activities (the number of seat belt citations, speeding tickets, other moving violations and warning per capita) per 10,000 population; and (vi) frequency of sobriety checkpoints (weekly, monthly, less than monthly, never).  Note that for independent variables, the enforcement activities were categorised into quintiles.  **Main dependent variables:** (i) DUI of alcohol at 3 BAC levels: BAC ≥0.01, ≥0.05, ≥0.08 g/dl.  **Adjusted variables:** characteristics of drivers: age, sex, race/ethnicity, whether passengers were in the car, seat belt usage and where the drivers were coming from (e.g., bar, restaurant, party). | None |
| 19 | Jia K, Fleiter J, King M, Sheehan M, Ma W, Lei J & Zhang J | 2016 | Alcohol-related driving in China: countermeasure implications of research conducted in two cities | To investigate knowledge on drunk driving law in two cities, China | China, city level | Police officers, drunk driving offenders and general drivers | Drink driving measures | **Study design and data collection methods:** The study applied mixed-methods. The study undertook three groups of study population. First, the study used semi-structured interviews among law enforcement officers to investigate problems that occur during enforcement. The study also conducted a cross-sectional survey among police officers to investigate their knowledge about existing regulations regarding drink driving measures. Second, the study used a cross-sectional survey among general motor vehicles drivers, which was based on a convenience sampling method to collect knowledge on drink driving law. A self-reported questionnaire was applied during the survey. Third, the study selected drink driving offenders to investigate their knowledge on existing regulations.  **Data analysis methods:** Chi-square test. | Used questionnaires | **Variables of interest: k**nowledge on BAC limit | None |
| 20 | Jia K, King M, Fleiter JJ, Sheehan M, Ma W, Lei J & Zhang J | 2016 | Drunk driving offenders’ knowledge and behavior in relation to alcohol-involved driving in Yinchuan and a comparison with Guangzhou, China | To investigate association between knowledge about amended law, exposure to enforcement, alcohol consumption, and alcohol involved driving behaviours | China, city level, Yinchuan | Drink driver offenders | Drink driving measures | **Study design and data collection methods:** Cross-sectional survey among drunk drivers who were in detention. A total of 106 offenders were recruited and interviewed by trained medical workers. The study also compared findings with previous a study using the same protocol, conducted in Guangzhou, China.  **Data analysis methods:** Multiple regression | Used questionnaires | **Main independent variables:** knowledge about alcohol-involved driving offenses: knowledge on drunk driving law, and knowledge on legal limit for drunk driving (right answer, wrong answer and don't know), and intensity of law enforcement.  **Main independent variables:** drink-driving behaviours.  **Adjusted variables:** socio-demographic characteristics, and alcohol consumption. | None |
| 21 | Lenk KM, Nelson TF, Toomey TL, Jones-Webb R & Erickson DJ | 2016 | Sobriety checkpoint and open container laws in the United States: associations with reported drinking-driving | To investigate association between state policy on sobriety checkpoints and open container law, law enforcement and self-report drink driving | USA, national level | General population | Drink driving measures | **Study design and data collection methods:** The study used a nationally representative survey among adults aged 18 years and over, and a cross-sectional survey among enforcement agencies using telephone interviews.  **Data analysis methods:** Multilevel modelling. | Used questionnaires | **Main independent variables:** existence of sobriety checkpoints and open container law, law enforcement of open container law (yes/no).  **Main dependent variables:** self-reported drink driving.  **Adjusted variables:** individual characteristics: sex, age, race, education, marital status, binge drinking. state level: population size, and total vehicle miles travelled. | None |
| 22 | Meesmann U, Martensen H & Dupont E | 2015 | Impact of alcohol checks and social norm on driving under the influence of alcohol (DUI) | To investigate association between alcohol checks, social norm and drink driving behaviors | Multi-countries, European countries | Drivers | Drink driving measures | **Study design and data collection methods:** The study applied a cross-sectional survey using face-to-face interviews among car drivers. The survey applied a quota sampling or random sampling method depending on the country.  **Data analysis methods:** Multilevel logistic regression. | Used questionnaires | **Main independent variables:** (i) perceived drink driving behaviours of friends (Likert scales); (ii) frequency of alcohol checks by police officers; (iii) perceived likelihood of getting caught for drink driving (Likert scales).  **Main dependent variables**: frequency of drink driving behaviours.  **Adjusted variables**: individual level (gender, age), and national level (legal alcohol limit, national level drunk driving of friends). | None |
| 23 | Morrison CN, Ferris J, Wiebe DJ, Peek-Asa C & Branas CC | 2019 | Sobriety checkpoints and alcohol-involved motor vehicle crashes at different temporal scales | To investigate association between sobriety checkpoints and alcohol-involved motor vehicle crashes at different temporal scales | USA, city level | General population | Drink driving measures | **Study design and data collection methods**: The study used registry data from two different sources. First, the alcohol-involved crash gathered from the Transport Injury Mapping Systems. Second, the number of sobriety checkpoints gathered from the Los Angeles Police Department. The two measures were estimated regarding days, weeks and months.  **Data analysis methods:** Autoregressive integrated moving average models. | Secondary data | **Main independent variables:** number of sobriety checkpoints.  **Main dependent variables:** alcohol-involved crashes.  **Adjusted variables:** none. | None |
| 24 | Nazif-Munoz JI, Quesnel-Vallée A & Van Den Berg A | 2015 | Did Chile’s traffic law reform push police enforcement? Understanding Chile’s traffic fatalities and injuries reduction | To investigate the impacts of law reform and police enforcement on traffic fatalities | Chile, national level | General population | Drink driving measures | **Study design and data collection methods:** The study used secondary data for both independent variables and dependent variables.  **Data analysis methods:** Ordinary least squares and robust random effects models | Secondary data sources | **Main independent variables:** (i) traffic reform using time period as cut point; (ii) number of police officers per population; (iii) police traffic enforcement measured by number of tickets processed by a local court divided by the number of police officers; (iv) road traffic instrumental mechanism generated by using law reform multiplied by police enforcement.  **Main dependent variables:** number of traffic fatalities.  **Adjusted variables:** oil price average, unemployment, percentage of young male population, alcohol consumption per capita, road traffic infrastructure (spending on public and private on public infrastructure). | None |
| 25 | Sanem JR, Erickson DJ, Rutledge PC, Lenk KM, Nelson TF, Jones-Webb R & Toomey TL | 2015 | Association between alcohol-impaired driving enforcement-related strategies and alcohol-impaired driving | To investigate the association between individual enforcement strategies, combined enforcement strategy, and drink driving behaviours | USA, national level | Enforcement agencies | Drink driving measures | **Study design and data collection methods:** The study employed a cross-sectional survey among enforcement agencies using telephone interview together with an online survey. The most knowledgeable on enforcement activities was selected.  **Data analysis methods:** Multilevel logistic regression. | Used questionnaires | **Main independent variables:** frequency of conducting sobriety checkpoints, conducting saturation patrols, enforcing open container laws, frequency of the use of media to publicise agency's enforcement effort and frequency of communicate or collaborate with local media.  **Main dependent variables:** alcohol-impaired driving.  **Adjusted variables:** gender, age, education, race and marital status. | None |
| 26 | Stringer RJ | 2019 | Policing the drunk driving problem: a longitudinal examination of DUI enforcement and alcohol related crashes in the U.S. (1985–2015). | To investigate association between DUI arrest and alcohol related crashes | USA, national level | Drunk drivers | Drink driving measures | **Study** **design and data collection methods:** The study used secondary data for both DUI arrests and traffic crashes. Time-series data from 1985-2015 was obtained.  **Data analysis methods:** Multilevel latent growth curve modelling | Secondary data sources | **Main independent variables:** DUI arrest.  **Main dependent variables:** alcohol-related crashes.  **Adjusted variables:** urban and rural areas, % bachelor degree or more, % population below poverty line, race, % population 18-24 years old, % population 25-34 years old, male and female ratio, total population, dry county, moist county, administrative license suspension, mean, self-report DUI, total vehicle miles travelled, total per-capita alcohol consumption, year. | None |
| 27 | Yao J, Johnson MB & Tippetts S | 2016 | Enforcement uniquely predicts reductions in alcohol-impaired crash fatalities | To investigate association between enforcement intensity and alcohol-impaired fatal crashes | USA, national level | General population | Drink driving measures | S**tudy design and data collection methods:** The study used secondary data.  **Data analysis methods:** Generalised linear mixed model | Secondary data sources | **Main independent variables:** driving under the influence of alcohol arrests per capita and funding of enforcement.  **Main dependent variables:** ratio of traffic fatality under influence of alcohol per number of traffic fatality without alcohol influence.  **Adjusted variables:** proportion of rural residents, median household income, proportion of high school graduates, proportion of college graduates, proportion of males/females, race/ethnicity, proportion of population aged 21-34 years. | None |
| **Multi-policy** | | | | | | | | | | | |
| 28 | Calvert C, Toomey T, Lenk K, Joshi S, Nelson T & Erickson D | 2020 | Variation in alcohol policy enforcement across urban and nonurban communities | To investigate differences in effort of law enforcement on alcohol policies between urban and rural communities | USA, national level | Enforcement agencies | Physical availability: underage drinking, social supply to minors, alcohol sales to minors and intoxicated persons, and open container law.  Drink-driving: sobriety checkpoints | S**tudy design and data collection methods:** The study used a cross-sectional survey to investigate enforcement activities among enforcement agencies. The agencies were selected based on multi-stage sampling methods. Telephone/online surveys were used to collect data from the most knowledgeable persons from different agencies.  **Data analysis methods:** Poisson regression | Used questionnaires | **Main independent variables**: urban, small town, suburban, urban areas.  **Main dependent variables**: conducting enforcement of law in seven main activities: (i) compliance checks to prevent illegal alcohol sales; (ii) social supply to minors; (iii) underage drinking; (iv) alcohol sales to intoxicated persons; (v) saturation patrols; (vi) sobriety checkpoints; and (vii) open container law.  **Adjusted variables**: percentage living under the poverty line, and percentage of population aged 18-30 years, size of agencies (large vs small; it is based on population size of the jurisdiction that agency served), and having officers assigned for enforcing alcohol control law. | None |
| 29 | Carragher N, Byrnes J, Doran CM & Shakeshaft A | 2014 | Developing an alcohol policy assessment toolkit: application in the western pacific | To assess national policies aimed at reducing alcohol consumption and related problems | Multi-countries, Western Pacific Region | Nine western Pacific countries | Physical availability: minimum purchasing age, alcohol server liability, government monopoly of alcohol retail sales, restriction of outlet density, restricted hours and days of alcohol sales.  Drinking context: community mobilisation, and mandatory training of bar staff.  Alcohol prices, alcohol advertising, drivers of motor vehicles | **Study design and data collection methods:** Ecological study using multiple sources of data. The data on enforcement was obtained from peer-review papers, WHO reports, governments and relevant websites.  **Data analysis method:** Pearson’s or Spearman's correlation coefficient. | Used questionnaires gathering information from relevant public health and government officials in the study country and secondary data in order to generate tool so-called the Toolkit for Evaluating Alcohol Policy Stringency and Enforcement-16. | **Main independent variables:** Alcohol Policy Scores, constructed from level of stringency, effectiveness rating, and level of enforcement among 16 policies.  **Main dependent variables:** alcohol consumption per capita.  **Adjusted variables:** crude associations. | Content validity, criterion validity and reliability |
| 30 | Casswell S, Meier P, MacKintosh AM, Brown A, Hastings G, Thamarangsi T, et al | 2012 | The International Alcohol Control (IAC) Study-Evaluating the Impact of Alcohol Policies | To investigate the association between alcohol policy environment and impact on alcohol consumption | England, Scotland, New Zealand, Thailand, and South Korea | Relevant stakeholders and general population | Physical availability, pricing and taxation, alcohol advertisement and drink-driving measures | **Study design and data collection methods:** The study applied mixed methods by using various types of methods for data collection: (i) document review of existing alcohol control policies; (ii) qualitative interviews, interviewing relevant stakeholders with knowledge of alcohol control; (iii) routinely collected data such as alcohol outlets; (iv) longitudinal survey among populations aged 16-65 years. Data analysis methods: Generalised estimating equation. | Used questionnaires | **Variables of interest:** Place and time of purchase, amounts purchased and price paid; ease of access to alcohol purchase; alcohol marketing measures; social supply; perceptions of alcohol affordability and availability and salience of price; perceptions of enforcement; people’s experiences with specific alcohol restrictions; support for policy and consumption (typical quantity, frequency using beverage and location-specific measures) | None |
| 31 | Casswell S, Morojele N, Williams PP, Chaiyasong S, Gordon R, Gray-Phillip G, et al | 2018 | The Alcohol Environment Protocol: A new tool for alcohol policy | To describe the implementation of alcohol control policies and level of enforcement regarding availability, marketing, and drink-driving measures, in high income countries, upper middle-income countries, and lower-middle income countries | Multi-countries: Scotland, New Zealand, St. Kitts and Nevis, Thailand, South Africa, Vietnam | Stakeholders who implemented alcohol control policies | Control of alcohol advertisement, control of physical availability, drink driving measures and pricing policy | **Study design and data collection methods**: The study used mixed methods, including document review, observational surveys, administration and commercial data, and a key informant interview.  **Data analysis method**: not applicable. | Used questionnaires | **Variables of interest:** restrictiveness of alcohol regulation, and key informants’ perception toward enforcement and compliance of regulations. | None |
| 32 | Erickson DJ, Rutledge PC, Lenk KM, Nelson TF, Jones-Webb R & Toomey TL | 2015 | Patterns of alcohol policy enforcement activities among local law enforcement agencies: a latent class analysis | To assess levels and patterns of alcohol policy enforcement activities among US local law enforcement agencies | USA, national level | Implementing agencies (law enforcement agencies) | Physical availability: (i) underage possession/consumption; (ii) underage provision; (iii) overservice of alcohol at alcohol establishment.  Drink-driving measure: impaired driving | **Study design and data collection methods:** Cross-sectional survey using a multi-stage sampling strategy.  The data was collected using self-administered questionnaires.  **Data analysis methods:** Using latent class to categorise groups of enforcement activities into low and high class (probability of enforcement activities less than 30% and more than 70%, respectively) and applied multinomial regression for multiple regression. | Used questionaries | **Main independent variables:** (i) number of officers per 1,000 population; (ii) whether any full-time offers assigned to enforce alcohol-related laws.  **Main dependent variables:** conducting enforcement activities.  **Adjusted variables:** (i) Agency characteristics: number of officers and whether those work full-time for enforcement of alcohol control policy law. Number of officers was based on a ratio/1000 resident in the agency's jurisdiction. (ii) Community characteristics: total population, percentage living in poverty, percentage of Black and Hispanic, region based on alcohol consumption level: dry, moderate or wet; and (iii) Perceptions on how common three problems are in their community: underage drinking, impaired driving, and overservice of alcohol (Likert scales). | None |
| 33 | Huckle T, Casswell S, Mackintosh AM, Chaiyasong S, Viet Cuong P, Morojele N, et al | 2018 | The International Alcohol Control Study: Methodology and implementation. | To report methods and implementation of the IAC Policy Study | Multi-countries, Australia, England, Scotland, New Zealand, St Kitts and Navis, Thailand, South Africa, Peru, Mongolia and Vietnam | Population aged 16-65 years old and key informants | Physical availability, pricing, promotion, and drink driving | **Study design and data collection methods:** The study applied mixed methods. There are two parts under IAC, including IAC survey, and Alcohol Environment Protocol, using document review, observation, and key informant interview.  IAC study used a random sample and some countries applied multi-stage sampling. Data collection was based on face-to-face interviews and telephone interviews depending on the country.   AEP used document review and key informant interviews. The sampling methods was based on purposive sampling.  **Data analysis methods:** Not applicable | Used questionnaires.  There were two tools used: (i) IAC, includes alcohol consumption, mediating policy variables (alcohol purchasing behaviours, respondents' usual travel-time to obtain alcohol and mode of travel, alcohol marketing measures, social supply, perceptions of alcohol affordability and availability, perceptions of enforcement, and perceptions of specific alcohol restrictions); (ii) AEP includes regulatory environment, knowledge on alcohol control among key stakeholders, enforcement, and compliance, perception of the alcohol environment, availability, marketing exposure. Levels of enforcement were measured on a Likert scale. | **Variables of interest:** perception towards different aspects (i.e., enforcements, compliance, and availability), patterns of alcohol consumption, and individual characteristics | None |
| 34 | Maclennan B, Kypri K, Connor J, Potiki T & Room R | 2016 | New Zealand's new alcohol laws: Protocol for a mixed-methods evaluation | To evaluate the effectiveness of the new laws in three aspects, including: (i) improving community input into licensing decisions; (ii) reducing the availability of alcohol in NZ communities; and (iii) reducing hazardous drinking and alcohol -related harm | New Zealand, national level | Five target groups of the study: (i) general population; (ii) Māori people or tribes; (iii) community groups who have taken actions on alcohol issues 4) territorial authorities 5) local government staff. | Physical availability: trading hours, outlet locations, alcohol licensing drink-driving, BAC limit among young groups | **Study design and data collection methods:** The study used mixed methods: (i) a national longitudinal survey; (ii) semi-structured interviews using a telephone survey, among Maoris, community groups, territorial authorities, and local governments; (iii) GIS to map alcohol outlets; (iv) secondary data (e.g., hospitals).  **Data analysis methods:** Using different analysis methods such as comparison of proportions, descriptive, logistic regression, negative binomial or Poisson regression. | Used questionnaires | **Main independent variables:** introduction of new laws.  **Main dependent variables:** (i) restrictiveness, number of local alcohol policies adopted/being developed by mid-2017; (ii) change in number of objections per license application; (iii) hazardous drinking; (iv) alcohol-related harms (i.e., second-hand and community amenity effects, late-night assaults per month and alcohol-involved traffic crashes per month); (v) community level: alcohol outlet density; (vi) changes in proportion of residents participating in local decision-making.  **Adjusted variables:** not mentioned. | None |
| 35 | Nilsson T, Leifman H & Andréasson S | 2015 | Monitoring local alcohol prevention in Sweden: Application of Alcohol Prevention Magnitude Measure (APMM) | To develop an APMM based on local data and to analyse the development of local alcohol prevention by using APMM. | Sweden, national level | Municipalities | Physical availability (age limits for alcohol, responsible beverage service), drink-driving measures, and brief intervention | **Study design and data collection methods:** The study used data from two main sources, including data from the former Swedish National Institute of Public Health, and web-based survey targeting all 290 municipalities in Sweden. The survey consisted off three areas, including inspections according to alcohol law and municipal alcohol and drug prevention.  **Data analysis methods:** Wilcoxon signed rank test | Used questionnaires and secondary data sources | The study included 37 indicators in five main components to create APMM, including: (i) staff and budget; (ii) policy; (iii) organising cooperation with authorities; (iv) supervision and licenses; and (v) licenses and activities.  Staff and budget: (i) number of staff positions for alcohol and/or drug prevention in the municipality; (ii) funding for alcohol and/or drug prevention is provided in the regular budget of the municipality; and (iii) a particular alcohol prevention coordinator is designated.  Policy: (i) municipal alcohol policy; (ii) years since alcohol policy was adopted; (iii) plan for implementation of the municipal alcohol policy; (iv) measurable goals in municipal alcohol policy; (v) plan for follow-up in municipal alcohol policy; (vi) particular funding set aside to perform activities in municipal alcohol policy; (vii) the municipality as an employer has an alcohol policy; (viii) The municipality has a policy for alcohol consumption in public places; (ix) NGOs are required to have an alcohol policy to be eligible for municipal funding; and (x) the municipality has a policy for alcohol prevention in elementary schools.  Organised cooperation with the authorities included three main sectors: (i) governmental organisations/authorities: police, health care, Swedish Transport Agency, other municipalities, county administrative board; (ii) businesses (restaurant owners, and entertainment businesses; (iii) NGOs (sport, temperance and religious organisations).  Supervision and license:  (i) number of regular alcohol licenses to the public per 10,000 inhabitants; (ii) proportion of licensed premises to the public closing later than 1 am; (iii) extent of supervision at licensed premises (public and private); and (iv) proportion of inspected grocery shops selling alcohol.  Activities: (i) arranging drug-free activities (e.g., school, dances and concerts); (ii) information to parents (e.g., leaflets and brochures); (iii) active work with media advocacy groups in order to increase awareness about alcohol and/or drugs; (iv) activities to limit illegal sales of alcohol to youth (e.g., information campaigns or police interventions); (v) activities to enforce age limits for alcohol sales; (vi) responsible beverage services; (vii) parental programs (with educated instructors) on alcohol and drugs in grades 6-9; (viii) group activities for children of substance misusing parents; (ix) activity for traffic sobriety, in addition to policy efforts; (x) brief intervention in primary health care. | Validity (i.e., Known-group technique), and reliability |
| 36 | Nilsson T, Norström T, Leifman H, Andréasson S, Guldbrandsson K & Allebeck P | 2020 | Effects of Local Alcohol Prevention Initiatives in Swedish Municipalities, 2006–2014 | To examine whether local alcohol prevention reduced consumption and alcohol-related harm in Swedish municipalities. | Sweden, national level | Municipal prevention coordinators or individuals in similar positions | Physical availability (age limits for alcohol, responsible beverage service), drink-driving measures and brief intervention | **Study design and data collection methods:** The study used multiple sources of data, based on Swedish municipalities. Data for the APMM originated from a yearly web-based survey regarding: (i) local drug prevention initiatives; and (ii) inspections pursuant to the *Alcohol Act* conducted by the Public Health Agency of Sweden. The survey was completed by municipal prevention coordinators or individuals in similar positions. Register data on licensed premises were also used in the APMM. The selection of municipalities was based on availability of data for at least the past nine years. Data on consumption and harm were based on data that was available at the local level.  **Data analysis methods:** fixed-effect model. | Used questionnaires and secondary data sources | **Main independent variables**: APMM, an index measured implementation at the local level.  **Main dependent variables:** alcohol sales (in litters per inhabitant), single-vehicle accidents between 10 pm and 5 am (per 10,000 inhabitant), police-reported assaults, outdoors, where the perpetrator was unknown to the victim per 10,000 inhabitants, treated patients and inpatient care with alcohol intoxication diagnosis as the primary and/or bi-diagnosis (morbidity), treated patients (morbidity), inpatient care with liver disease as the primary diagnosis (morbidity), deceased with explicit alcohol diagnosis as the underlying and/or contributory cause of death (mortality).  **Adjusted variables:** median income, unemployment rate, post-secondary education, and population size.  The study included 37 indicators in five main components to create the APMM, including: (i) staff and budget; (ii) policy; (iii) organising cooperation with authorities; (iv) supervision and licenses; and (v) licenses and activities. See details of each indicator in the study from Nilsson T, Leifman H & Andréasson S, 2015. | Validity (i.e., known-group technique), and reliability |
| 37 | Paschall MJ, Grube JW, Thomas S, Cannon C & Treffers R | 2012 | Relationships between local enforcement, alcohol availability, drinking norms, and adolescent alcohol use in 50 California cities | To investigate relationships between local alcohol policies, enforcement, alcohol outlet density, adult alcohol use, and underage drinking in 50 California cities. | USA, city level | 50 cities in California and youth | (i) control of physical availability: conditional use permit required for new establishments selling or serving alcohol (e.g., designating hours of operation; approved selling or serving comply with minimum operational standards; public drinking prohibition; special outdoor events policies; (ii) harm reduction policies: social host policies; (iii) alcohol advertisement: outdoor advertising/billboards of alcoholic beverages prohibited; window advertising of alcoholic beverages prohibited. | **Study design and data collection methods:** The study included different data sources: (i) youth survey based on telephone interviews (adolescent alcohol use and heavy drinking, perceived availability of alcohol, perceived enforcement of underage drinking laws, perceived acceptability of alcohol use); (ii) adult drinking survey, telephone interviews (adult drinking); (iii) secondary data sources (population and outlet data, funding of enforcement activities).  **Data analysis methods:** Multi-level linear analysis. | Used questionnaires and secondary data | **Main independent variables:** (i) local enforcement activities, used funding on activities as proxy; (ii) stringency and comprehensiveness of each type of ordinance, city received +1 if had the relevant type of law existed and a 0 if no such law existed.  **Main dependent variables:** adolescent alcohol use and heavy drinking.  **Adjusted variables**:City level: (i) alcohol outlet density, outlets per roadway mile in each city; (ii) adult alcohol use.  Individual level: (i) perceived availability of alcohol (Likert scales); (ii) perceived enforcement of underage drinking laws by asking about the likelihood of being caught by police in six situations (Likert scales); (iii) perceived acceptability of alcohol use (Likert scales); (iv) age; (v) gender; (vi) race/ethnicity | Reliability |
| 38 | Paschall MJ, Lipperman-Kreda S & Grube JW | 2014 | Effects of the local alcohol environment on adolescents’ drinking behaviours and beliefs | To investigate associations between characteristics of the local alcohol environment and adolescent alcohol use and beliefs in 50 California cities | USA, state level | Adolescents, 13-17 years old | Physical availability control: conditional use permit required for new establishment selling or serving alcohol, deemed approved requirements that pre-existing establishment selling or serving alcohol comply, s, public drinking prohibition, responsible beverage service training required for staff of establishments selling or serving alcohol, social host policies, special outdoor events policies governing alcohol services (street fair) alcohol advertisement: outdoor advertising/billboard, and window advertising of alcohol beverage prohibition | **Study design and methods of data collection:** The study used data from various sources and one source was a cohort study design: (i) youth survey, 3 waves using computer-assisted telephone interviews to investigate adolescent alcohol use, heavy drinking, perceived availability of alcohol, perceived enforcement of underage drinking laws, perceived approval of alcohol use,and youth demographics; (ii) online survey and interviews with city clerks for local alcohol policy data; (iii) other secondary data sources were used for local enforcement activities, bar and density; (iv) adult survey using telephone interview on alcohol use.  **Analysis method:** Multilevel logistic regression. | Used questionnaires and secondary data | **Main dependent variables:** Alcohol environment: (i) stringency and comprehensiveness of each type of ordinance; (ii) local enforcement activities, used funding on activities as proxy; (iii) alcohol outlet density, outlets per roadway mile in each city; (iv)adult alcohol use.  **Main dependent variables:** past year alcohol use and past year heavy drinking among youth.  **Adjusted variables:** City level: city demographics. Individual level: perceived alcohol availability (Likert scales), perceived enforcement of underage drinking laws (Likert scales), perceived parental approval of alcohol use (Likert scales). | Reliability |
| 39 | Randerson S, Casswell S & Huckle T | 2018 | Changes in New Zealand’s alcohol environment following implementation of the *Sale and Supply of Alcohol Act* (2012) | To assess the impact of the *Sale and Supply of Alcohol Act* 2012 | New Zealand, national level | Police officers, liquor licensing inspectors, and public health officers | Physical availability: social supply to minors, trading hours, and supply to intoxicated persons. Marketing: point-of-sale marketing, and drink driving | **Study design and data collection methods:** The study applied mixed methods. Key informant interview for rating of law enforcement and compliance and also provided reasons for rating. Key informants were purposively selected, including those involved in monitoring licensed environments, enforcing alcohol regulations, checking license conditions, and reviewing alcohol license applications.  **Data analysis methods:** Ordered logistic regression and thematic analysis. | Used questionnaires | **Variables of interest:** Compliance and enforcement level of policies.  Likert scale questions were used to assess level of enforcement and compliance in different policy domains: social supply to minors, trading hours, supply of alcohol to intoxicated persons, point-of-sale marketing and drink driving. | None |

*Not reported/did not assess validity or reliability.

ANOVA, analysis of variance; APMM, Alcohol Prevention Magnitude Measure; BAC; blood alcohol concentration; CIZ, cumulative impact zone; DUI, driving under influence; IAC, International Alcohol Control Policy; NGO, non-government organisation; NRS, National Road Side Survey; WHO, World Health Organization.
